# High-Temperature Thermodynamic Functions for Zirconium and Unsaturated Zirconium Hydrides

**DOI:** 10.6028/jres.067A.043

**Published:** 1963-10-01

**Authors:** Thomas B. Douglas

## Abstract

Giving greatest weight to the experimentally measured highest decomposition pressures and the enthalpies in one-phase fields, thermodynamically interconsistent integral and differential enthalpies (heat contents), heat capacities, entropies, and Gibbs free energies are derived for the crystalline one- and two-phase fields of the zirconium-hydrogen system for all stoichiometric compositions from Zr to ZrH_1.25_ and over the temperature range 298.15 to 1,200 °K. These properties are derived in analytical form, and in most cases are represented by numerical equations, with tabulation for zirconium and H/Zr atom ratios of 0.25, 0.50, 0.57, 0.75, 1.00, and 1.25. Most of the unique phase-field boundaries which are consistent with the derived properties are located and are compared with those previously reported. In the Zr-H system the enthalpies are shown to relate certain properties at different compositions as well as at different temperatures. Some of the various data show good interconsistency, while others reveal discrepancies which are discussed critically.

## 1. Introduction

Among transition-metal hydrides, the zirconium-hydrogen system has been studied very extensively in recent years because of the practical importance of and theoretical interest in the system. Zirconium hydrides are potentially valuable thermalneutron moderators which in some cases can reach elevated temperatures without attaining excessive pressures. The wide ranges of composition, interrupted by lattice changes and marked miscibility gaps, offer a fruitful system for studying the structure of interstitial solid hydrides.

Experimental data on the thermodynamic and other physical properties of the system have been published by a number of investigators. Some of these have measured equilibrium pressures of hydrogen in certain ranges of temperature and composition [1, 2, 3, 4, 5, 6, 7, 8].^1^ The rates of diffusion of hydrogen in the metal have been studied [9, 7]. X-ray [10] and neutron-diffraction [11] studies have given information on the zirconium- and hydrogen-atom spacings, respectively. Measurements have been made of thermal conductivity [12] and thermal expansion [12, 13, 14]. The enthalpies or heat capacities have been measured for the “saturated” composition ZrH_2_ at low temperatures [15], and for several “unsaturated” compositions at high temperatures [16, 12]. Many of these data have served to outline the phase diagram of the system by a variety of methods, as the author showed in a previous paper [17]. Attention may also be called to some of the publications which have been particularly directed toward reviewing these properties, correlating them thermodynamically, and discussing their bearing on an interpretation of the structures of these phases [4, 15, 17, 18, 19].

In this paper is presented a consistent set of the common integral and partial thermodynamic functions of the zirconium-hydrogen system based on some of the aforementioned data. These functions were derived from the data without recourse to extra-thermodynamic considerations except, incidentally, such general and well-established ones as the laws of dilute solutions. The composition range covered is from Zr to ZrH_1.25_, and the temperature range from 298.15 to 1,200 °K. (Though thermodynamic data are available for higher temperatures [1] and higher hydrogen contents [2,3,5,8,10], these were omitted from consideration because of their generally inferior precision or lack of agreement.) The coexistence of phases in equilibrium is in a sense accidental, and the theoretical interest in the system therefore centers mainly in the properties of individual phases. But from a practical standpoint the equilibrium reproportionation of phases with temperature change of a given overall composition occurs fairly rapidly in some cases and is of interest; for this reason properties have been evaluated for two-phase as well as for one-phase regions. As will be pointed out, the available data show excellent interconsistency in some ranges of composition and temperature. In other regions considerable discrepancies were found which introduce considerable uncertainty into the adopted thermodynamic functions, particularly in two-phase regions. It will be evident that the requirement of thermodynamic consistency for a multicomponent system imposes an often unsuspected multitude of restrictions on the relations of the properties for neighboring compositions and temperatures, particularly near the center of the region under consideration in the phase diagram. This is true even for a two-component system which, like the present one, has a phase diagram which is only moderately complex.

The opinion has been expressed [6] that the purity of the zirconium used in the various experimental studies on the zirconium-hydrogen system was not sufficiently high for the results of a full thermodynamic analysis to be of any great significance. The present paper will illustrate that such an analysis can be rather laborious, and that, in the present case, it reveals numerous inconsistencies in the data which lend some weight to that opinion. Nevertheless, the author feels that only by a thermodynamic correlation of this type can the interconsistencies of the various data be tested, a better idea of their reliability be gained, additional properties of the system be derived, and the need for additional experimental work in certain areas be suggested. However, to the extent that the data are inconsistent, an adopted correlation will necessarily be somewhat arbitrary, particularly since the theoretical number of different ways to weight the numerous interrelated data is found to be quite large. In another sense, one can be arbitrary in deciding which borderline theoretical relationships are sufficiently valid to impose on the correlation.

**Figure 1:**
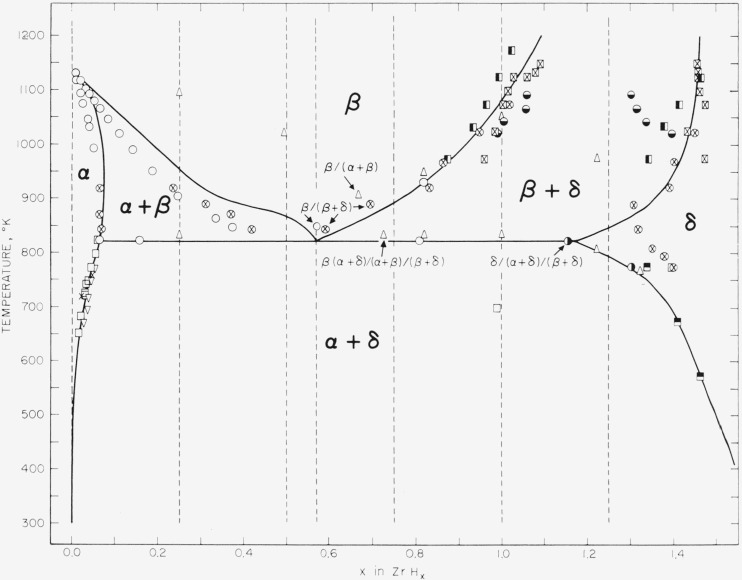
Phase diagram (partial) of the zirconium-hydrogen system. (Observed points on phase boundaries are shown as follows. *From equilibrium hydrogen pressures:* ◒ Hall, Martin, and Rees [2]; □ Gulbransen and Andrew [4]; ☒ Edwards, Levesque, and Cubicciotti [5]; ○ Ells and McQuillan [6]; X Mallett and Albrecht [7]; Ⓧ LaGrange, Dykstra, Dixon, and Merten [8] *From hydrogen diffusion:* ∇ Schxvartz and Mallett [9]. *From X-ray diffraction:* ∆ Vaughan and Bridge [10]. *From thermal expansion:* ◧ Beck [12]; 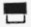 Espagno, Azou, and Bastien [14]. *From enthalpy:* ◒ Douglas [17]. The interconsistent thermodynamic functions formulated in this paper correspond to the solid-curve boundaries, and are tabulated along the dotted lines.)

The phase diagram of the zirconium-hydrogen system in the region treated in this paper is shown in figure 1. The symbols *α*, *β*, and *δ*^2^ refer to single-phase regions where the zirconium lattice is close-packed hexagonal, body-centered cubic, and face-centered tetragonal,^3^ respectively. The points shown on the diagram are those on phase-field boundaries as found by various investigators, and the solid boundaries are those to which the thermodynamic properties adopted in this paper correspond. The majority of these properties are presented in analytical form for the continuous ranges of temperature and composition, and are tabulated along the dotted vertical lines shown in the figure.

## 2. Thermodynamic Preliminaries

### 2.1. Conventions

The unit of energy used throughout is the defined thermochemical calorie (=4.1840 j), and all temperatures are in deg K. All extensive properties of a zirconium hydride are evaluated for “one mole” defined as containing a total of 1 g-atom of Zr and *x* g-atoms of H,^4^ even when one mole so defined is composed of two or more phases in equilibrium. The components are taken to be alpha-zirconium (at *all* temperatures, to preserve continuity into the small 64° temperature interval covered where beta-zirconium is more stable) and “normal” molecular hydrogen in the usual standard state of the ideal gas at 1 atm.^5^

The symbols in common usage have been adopted so far as possible. Those not specifically defined are:

*a_i_*: Thermodynamic activity of component *i.* (*a*_2_ is taken equal to the equilibrium pressure of H_2_(g) in atm.)
*b, c*, *b+c*: Generalized notation for particular one- or two-phase fields. (In the absence of specific designation, the phase field is implied as any one, or the particular one under discussion.)
*C_p_*: Heat capacity at constant pressure and constant overall composition (cal mole^−1^ deg K^−1^).
*f*: “Of formation” (usually from the components in their standard states).
*G*: Absolute Gibbs free energy (cal mole^−1^).
*H*: Absolute enthalpy (heat content) (cal mole^−1^).
*H*′: “Intrinsic enthalpy” (A*H* of the reaction of eq (30)).
H_2_(g): Hydrogen gas.
*i* (subscript): A particular component (either).
ln: Natural logarithm.
log: Logarithm to the base 10.
*R*: The molar gas constant (= 1.98725 cal mole^−1^ deg K^−1^).
*S*: Absolute entropy (cal mole^−1^ deg K^−1^).
*T*: Absolute temperature (deg K, Int. Temp. Scale of 1948).
*x*: Atoms of H per atom of Zr (in the overall composition when two phases are present).
*x_b_* and *x_c_*: Define the compositions of the two coexistent phases ${\mathrm{ZrH}}_{x_{b}}$ and ${\mathrm{ZrH}}_{x_{c}}$ which lie on the phase-field boundaries of the (*b* + *c*) field.
*Y*: Generalized notation for one of *H*, *C_p_, S*, or *G.*
${\bar{Y}}_{i}$: artial molal *Y* with respect to component *i.*
*α, β, δ*: Types of single phases of ZrH*_x_*. (See fig. 1.)
(*α* + *β*), etc.: Two-phase field, or an overall composition lying therein.
*α*/(*α*+*β*), etc.: Boundary between the *α* and (*α*+*β*) fields, etc.
1 (subscript): The component zirconium, Zr.
2 (subscript): The component molecular hydrogen, H_2_.

A numerical subscript or superscript other than 1 or 2: When ≦2, a particular value of *x;* when >2, a particular value of *T.* (The absence of such subscripts implies general values of *x* and *T*, or a particular set under discussion.)

^0^ or °(superscript) : In the standard state (see text); deg temperature.

### 2.2. General Thermodynamic Relations

Several well-known thermodynamic relations frequently used in subsequent derivations or calculations are assembled here. These have been adapted to the specialized definition of one mole of Zr, H_2_, and ZrH*_x_* stated above.

Total properties are often conveniently derived using

$$C_{p}^{{}^{\circ}}={\left[\partial \left(H{}^{\circ}-H_{298.15}^{{}^{\circ}}\right)/\partial T\right]}_{x};$$ (1)

$${\left(\partial S{}^{\circ}/\partial T\right)}_{x}=C_{p}^{{}^{\circ}}/T;$$ (2)

$$-\left(G{}^{\circ}-H_{298.15}^{{}^{\circ}}\right)/T=S{}^{\circ}-\left(H{}^{\circ}-H_{298.15}^{{}^{\circ}}\right)/T.$$ (3)

(Equation (3) has been used throughout this paper to obtain the free-energy function.) A relation similar to eq (3) applies to the relative partial molal properties:

$$\left({\bar{G}}_{i}-G_{i}^{{}^{\circ}}\right)-T=\left({\bar{H}}_{i}-H_{i}^{{}^{\circ}}\right)/T-\left({\bar{S}}_{i}-S_{i}^{{}^{\circ}}\right).$$ (4)

The total and partial properties are related by

$$Y{}^{\circ}={\bar{Y}}_{1}+\frac{1}{2}x{\bar{Y}}_{2}$$ (5)

and

$${\bar{Y}}_{2}=2{\left(\partial Y{}^{\circ}/\partial x\right)}_{T},$$ (6)

integration of the latter between two compositions *x*′ and *x*″ giving

$$Y_{x^{\prime \prime }}^{{}^{\circ}}-Y_{x^{\prime }}^{{}^{\circ}}=\frac{1}{2}\int_{x^{\prime }}^{x^{\prime \prime }}{\bar{Y}}_{2}dx.$$ (7)

From eqs (5) and (6)

$${\bar{Y}}_{1}=Y{}^{\circ}-x{\left(\partial Y{}^{\circ}/\partial x\right)}_{T}.$$ (8)

The Gibbs-Duhem equation takes the form

$${\left[\partial \left({\bar{Y}}_{1}-Y_{1}^{{}^{\circ}}\right)/\partial x\right]}_{T}+\frac{1}{2}x{\left[\partial \left({\bar{Y}}_{2}-Y_{2}^{{}^{\circ}}\right)/\partial x\right]}_{T}=0.$$ (9)

By the definition of activity

$$\left({\bar{G}}_{i}-G_{i}^{{}^{\circ}}\right)/T=R\;\ln\;a_{i}.$$ (10)

The variation of the partial molal free energy with temperature is given by

$${\left[\partial \left(\frac{{\bar{G}}_{i}-G_{i}^{{}^{\circ}}}{T}\right)/\partial T\right]}_{x}=-\left({\bar{H}}_{i}-H_{i}^{{}^{\circ}}\right)T^{2}.$$ (11)

From eq (5) follows the standard heat, entropy, and free energy of formation at any temperature. Since

$$\Delta Yf{}^{\circ}=Y{}^{\circ}-\left(Y_{1}^{{}^{\circ}}+\frac{1}{2}xY_{2}^{{}^{\circ}}\right),$$ (12)

we have

$$\Delta Hf{}^{\circ}=\left({\bar{H}}_{1}-H_{1}^{{}^{\circ}}\right)+\frac{1}{2}x\left({\bar{H}}_{2}-H_{2}^{{}^{\circ}}\right);$$ (13)

$$\Delta Sf{}^{\circ}=\left({\bar{S}}_{1}-S_{1}^{{}^{\circ}}\right)+\frac{1}{2}x\left({\bar{S}}_{2}-S_{2}^{{}^{\circ}}\right);$$ (14)

$$\Delta Gf{}^{\circ}=\left({\bar{G}}_{1}-G_{1}^{{}^{\circ}}\right)+\frac{1}{2}x\left({\bar{G}}_{2}-G_{2}^{{}^{\circ}}\right).$$ (15)

The molal enthalpy, heat capacity, entropy, or free energy in a two-phase field is the sum of those of the component (or “terminal”) phases:

$$Y_{x}^{b+c}=\left[\left(x_{c}-x\right)/\left(x_{c}-x_{b}\right)\right]Y^{b}+\left[\left(x-x_{b}\right)/\left(x_{c}-x_{b}\right)\right]Y^{c}.$$ (16)

In the present case it is particularly simple to relate two common alternative conditions determining this separation into the two terminal phases. One commonly stated obvious condition is that a straight line be tangent to the *continuous* free-energy isotherm at *x_b_* and *x_c_* and lie lower than the isotherm between *x_b_* and *x_c_.* Hence

$${\left(dG/dx\right)}_{x_{b}}={\left(dG/dx\right)}_{x_{c}},$$ (17)

and hence from eq (6)

$${\left({\bar{G}}_{2}\right)}_{x_{b}}={\left({\bar{G}}_{2}\right)}_{x_{c}}\equiv {\bar{G}}_{2}.$$ (18)

From eqs (5) and (18)

$$G_{x_{b}}={\left({\bar{G}}_{1}\right)}_{x_{b}}+\frac{1}{2}x_{b}{\bar{G}}_{2}$$ (19)

and

$$G_{x_{c}}={\left({\bar{G}}_{1}\right)}_{x_{c}}+\frac{1}{2}x_{c}{\bar{G}}_{2},$$ (20)

whose difference gives

$$\left[G_{x_{c}}-G_{x_{b}}\right]/\left[x_{c}-x_{b}\right]=\left[{\left({\bar{G}}_{1}\right)}_{x_{c}}-{\left({\bar{G}}_{1}\right)}_{x_{b}}\right]/\left[x_{c}-x_{b}\right]+\frac{1}{2}{\bar{G}}_{2}.$$ (21)

From the condition of tangency and eq (6) the first and third terms of eq (21) are equal, and hence

$${\left({\bar{G}}_{1}\right)}_{x_{b}}={\left(G_{1}\right)}_{x_{c}}.$$ (22)

Equations (18) and (22) are the more commonly stated conditions for phase coexistence. The condition that the tangent line lie lower than the metastable free-energy curve between requires that at each point of tangency *d^2^G/dx*^2^ be positive, and from equation (6) this is true if at each of these points $d{\bar{G}}_{2}/dx$ is positive—i.e., if the activity of hydrogen in each phase increases isothermally with increasing hydrogen content.

## 3. Basic Data Used

The direct measurements of the equilibrium pressures of hydrogen for the system extend from 650 to 1,373 °K and over the composition range from near *x*=0 to *x*>1.7. These data are sufficient to determine a set of the common energetic thermodynamic properties of the system in this range. However, these sets of data overlap considerably and with varying degrees of disagreement. In addition, those of the precise enthalpy data which may be considered valid, while they alone neither establish activities nor their temperature derivatives, do impose relations among the activity values at a given composition and different temperatures and also, as will be presently shown, among the temperature derivatives of activity for different compositions.

Enthalpy measurements relative to *T*= 273 were made by Douglas and Victor [16] (usually at temperatures spaced at 50- or 100-deg intervals) up to *T*= 1,173 for the compositions *x*=0, 0.324, 0.556, 0.701, 0.999, and 1.071. These measurements were all made by the drop method, and in all cases involved fairly rapid cooling in the low-temperature two-phase *(α*+*δ*) field, where composition equilibrium corresponds to phase reproportionation (see fig. 1). However, evidence will be shown in section 5 that phase equilibrium was approximately maintained in this field in these measurements. It would also be expected that composition equilibrium was attained for temperature intervals beginning in the one-phase field involved (the *β* field), and this was indicated by the high precision, regularity, and freedom from hysteresis found for most of these particular measurements (see sec. 6).

In view of the above facts, it was decided to construct the adopted consistent set of thermodynamic functions for the system largely by giving greatest weight to the hydrogen-activity data at the higher temperatures and to use the enthalpy data considered valid in order to extend the properties so derived to other temperatures and other compositions. The measurement of the highest equilibrium pressures is ordinarily subject to the smallest percentage errors, and this is often true with respect to the effects of small amounts of interfering impurities in the samples.

The hydrogen-activity data so chosen as a basis were those reported graphically by LaGrange, Dykstra, Dixon, and Merten [8] at *T*= 1,073.2 and 13 compositions (*x*=0.05 to 1), and those reported in tabular form by Edwards, Levesque, and Cubicciotti [5] for values of *x* of 0.650, 0.733, 0.815, and 0.894 and at 8 temperatures over the range *T*= 1,023 to 1,173. (These points all lie in the *β* field.) The values of the latter authors were first all reduced to *T*= 1,073.2 by using their smoothed temperature coefficients in the usual way. A reasonably good fit to these data was found to be given by

$$\alpha_{2\;\left(1073.2\right)}^{\beta }=0.06084x^{1.36}+0.11251x^{8.33},$$ (23)

or

$${\left[\left({\bar{G}}_{2}^{\beta }-G_{2}^{{}^{\circ}}\right)/T\right]}_{1073.2}=6.22313\;\log\;x+4.5758\;\log\;\left(1+1.8493x^{6.97}\right)-5.56334.$$ (24)

The agreement with the values fit (which vary from 0.002 to 0.16 atm) is shown in table 1; over the range *x*=0.19 to 1 it averages 2 percent, which is within the precision of the measurements. Calculations of cycles of entropy and zirconium-activity which are presented in section 7 depend in part on the accuracy of eq (23) (or (24)), and show excellent consistency.

It is appropriate to examine eq (23) in the light of the fact that several observers have found a proportionality, at constant temperature and only moderately high hydrogen concentrations, between the activity *a*_2_ and the *square* of the hydrogen concentration (“Sieverts’ law”), which corresponds to an activity coefficient of *atomic* hydrogen independent of composition. Up to *x*=0.5 the last term in eq (23) contributes less than 2 percent of the total, so that the power of *x* is not 2 but closer to 1. Sieverts and Roell [1] measured hydrogen-pressure isotherms for beta-zirconium at 1,073° and 1,373 °K, and though their data show inferior precision compared with that of the more recent measurements fitted above, their results show an approximate proportionality of *a*_2_ to *x*^2^ at the higher temperature but not at the lower. It is easy to show, by substituting from eq (10) into eq (11), replacing *a*_2_ by a single term proportional to *x^n^*, and then differentiating partially with respect to *x*, that the power *n* is independent of *T* if and only if $\left({\bar{H}}_{2}-H_{2}^{{}^{\circ}}\right)$ does not vary with *x.* It will in fact be found that although $\left({\bar{H}}_{2}-H_{2}^{{}^{\circ}}\right)$ near 1,073 °K is approximately independent of *x* at the higher values of *x*, it is far from being so for x<0.5. The sign and magnitudes of $\partial \left({\bar{H}}_{2}-H_{2}^{{}^{\circ}}\right)/\partial x$ in the latter region correspond to an approach to the *x*^2^ law as the temperature rises, but for lack of enough precise data, no serious effort was made to interpolate the pressure data over the range 1,073 to 1,373 °K.

The manner of using the enthalpy data will next be considered. In the first place, it is apparent from figure 1 that in all these enthalpy measurements for the five hydride compositions the sample was cooled into the (*α+δ*) field at 298 °K. In section 5 the following value will be derived (from eq (90)):

$${\left({\bar{H}}_{1}^{\alpha +\delta }-H_{1}^{{}^{\circ}}\right)}_{298}=4.$$ (25)

This value is very small because at this temperature the composition range of the *α* field is very small. We may now consider ${\left({\bar{H}}_{2}^{\alpha +\delta }-H_{2}^{{}^{\circ}}\right)}_{298}$. Applying eq (6) to the absolute enthalpy at any temperature and also at 298 °K, and subtracting the two, one may write

$$\left({\bar{H}}_{2}-H_{2}^{{}^{\circ}}\right)=2\left[\partial \left(H{}^{\circ}-H_{298}^{{}^{\circ}\left(\alpha +\delta \right)}\right)/\partial x\right]+{\left({\bar{H}}_{2}^{\left(\alpha +\delta \right)}-H_{2}^{{}^{\circ}}\right)}_{298}-\left(H_{2}^{{}^{\circ}}-H_{2\left(298\right)}^{{}^{\circ}}\right).$$ (26)

^6^The application of eq (6) to eq (16) shows that relative partial molal functions in a two-phase field at a given temperature (including ${\left({\bar{H}}_{2}^{\left(\alpha +\delta \right)}-H_{2}^{{}^{\circ}}\right)}_{298}$) are independent of *x.* Consequently, if data at one temperature and composition are available for evaluating ${\left({\bar{H}}_{2}^{\left(\alpha +\delta \right)}-H_{2}^{{}^{\circ}}\right)}_{298}$ from eq (26), then the same equation, with this value substituted, may be used to evaluate $\left({\bar{H}}_{2}-H_{2}^{{}^{\circ}}\right)$ at other temperatures and compositions where ${\left[\partial \left(\bar{H}{}^{\circ}-H_{298}^{{}^{\circ}}\right)/\partial x\right]}_{T}$ can be evaluated by suitable interpolation.

Plots of the hydrogen-pressure data of Edwards et al. [5] for the four compositions from *x*= 0.650 to *x*= 0.894, used above, gave without trend almost identical values which averaged ${\left({\bar{H}}_{2}^{\beta }-H_{2}^{{}^{\circ}}\right)}_{1123}=-40,500$. Ells and McQuillan [6] measured $\alpha_{2}^{\beta }$ at one high concentration of hydrogen (*x*=0.66) from approximately 875 to 1,035 °K, leading to a value which when corrected to 1,123 °K by the enthalpy data gave ${\left({\bar{H}}_{2}^{\beta }-H_{2}^{{}^{\circ}}\right)}_{1123}=-37,500$. A weighted mean was adopted:

$${\left({\bar{H}}_{2}^{\beta }-H_{2}^{{}^{\circ}}\right)}_{1123,0.7}=-37,500.$$ (27)

For many calculations in this paper the relative enthalpy of molecular hydrogen gas has been computed from the empirical equation

$$H_{2}^{{}^{\circ}}-H_{2\left(298\right)}^{{}^{\circ}}=6.660T+2.678\left(10^{-4}\right)T^{2}-12000T^{-1}-1969,$$ (28)

which between 298 and 1,200 °K agrees within an average of about 3 cal mole^−1^ with the accurate values [20]. Equation (28) gives $H_{2\left(1123\right)}^{{}^{\circ}}-H_{2\left(298\right)}^{{}^{\circ}}=5837$. From the enthalpy of the pertinent *β* zirconium hydrides as formulated in section 6 (eq (92)), $2\left[\partial \left(H_{\left(1123\right)}^{{}^{\circ}}-H_{\left(298\right)}^{{}^{\circ}}\right)/\partial x\right]=7815$. Using eq (27) and these values, eq (26) gives

$${\left({\bar{H}}_{2}^{\alpha +\delta }-H_{2}^{{}^{\circ}}\right)}_{298}=-41,478.$$ (29)

Equation (29) has an uncertainty of at least 1,000 cal mole^−1^, but the additional significant figures serve to maintain consistency in many of the subsequently derived values.

An equation equivalent to eq (26) in integral form may be derived which is more general because it indicates the energy difference between two zirconium hydrides where both *x* and *T* may differ finitely. If we consider a system of 1 g-atom of zirconium and 2 of hydrogen, and arbitrarily define the reference state as the two free elements in their standard states at a temperature of 298 °K, the increase in enthalpy when the temperature or chemical composition or both change may be called the "intrinsic enthalpy” (*H*′).^7^ The process is

$$\mathrm{Zr}\left(298{}^{\circ}K\right)+H_{2}\left(g\right)\left(298{}^{\circ}K\right)={\mathrm{ZrH}}_{x}\left(T\right)+\left(1-\frac{1}{2}x\right)H_{2}\left(g\right)\left(T\right),$$ (30)

and

$$H^{\prime }=H{}^{\circ}+\left(1-\frac{1}{2}x\right)H_{2}^{{}^{\circ}}-H_{1\left(298\right)}^{{}^{\circ}}-H_{2\left(298\right)}^{{}^{\circ}}.$$ (31)

From eq (13)

$$H_{298}^{{}^{\circ}\left(\alpha +\delta \right)}-H_{1\left(298\right)}^{{}^{\circ}}-\frac{1}{2}xH_{2\left(298\right)}^{{}^{\circ}}={\left({\bar{H}}_{1}^{\alpha +\delta }-H_{1}^{{}^{\circ}}\right)}_{298}+\frac{1}{2}x{\left({\bar{H}}_{2}^{\alpha +\delta }-H_{2}^{{}^{\circ}}\right)}_{298}.$$ (32)

Adding eqs (31) and (32),

$$H^{\prime }=\left(H{}^{\circ}-H_{298}^{{}^{\circ}\left(\alpha +\delta \right)}\right)+{\left({\bar{H}}_{1}^{\alpha +\delta }-H_{1}^{{}^{\circ}}\right)}_{298}+\frac{1}{2}x{\left({\bar{H}}_{2}^{\alpha +\delta }-H_{2}^{{}^{\circ}}\right)}_{298}+\left(1-\frac{1}{2}x\right)\left(H_{2}^{{}^{\circ}}-H_{2\left(298\right)}^{{}^{\circ}}\right).$$ (33)

This result may be applied to two compositions ${\mathrm{ZrH}}_{x}^{\prime }$ and ${\mathrm{ZrH}}_{x}^{\prime \prime }$ at *T*′ and *T*″ respectively. Thus for

$${\mathrm{ZrH}}_{x^{\prime }}\left(T^{\prime }\right)+\frac{1}{2}\left(x^{\prime \prime }-x^{\prime }\right)H_{2}\left(g\right)\left(T^{\prime }\right)={\mathrm{ZrH}}_{x^{\prime \prime }}\left(T^{\prime \prime }\right)$$ (34)

eq (33) leads to

$$\Delta H={\left(H{}^{\circ}-H_{298}^{{}^{\circ}\left(\alpha +\delta \right)}\right)}_{x^{\prime \prime }}-{\left(H{}^{\circ}-H_{298}^{{}^{\circ}\left(\alpha +\delta \right)}\right)}_{x^{\prime }}+\frac{1}{2}\left(x^{\prime \prime }-x^{\prime }\right){\left({\bar{H}}_{2}^{\alpha +\delta }-H_{2}^{{}^{\circ}}\right)}_{298}-\frac{1}{2}\left(x^{\prime \prime }-x^{\prime }\right)\left(H_{2\left(T^{\prime }\right)}^{{}^{\circ}}-H_{2\left(298\right)}^{{}^{\circ}}\right).$$ (35)

In equation (26), (33), or (35), $\left(H_{2}^{{}^{\circ}}-H_{2\left(298\right)}^{{}^{\circ}}\right)$ and ${\left({\bar{H}}_{2}^{\alpha +\delta }-H_{2}^{{}^{\circ}}\right)}_{298}$ can be evaluated from eqs (28) and (29) respectively. Partial differentiation of eq (33) with respect to *x* and substitution from eq (6) show that

$${\left(\partial H^{\prime }/\partial x\right)}_{T}=\frac{1}{2}\left({\bar{H}}_{2}-H_{2}^{{}^{\circ}}\right).$$ (36)

With the help of eq (32), eq (5) may be put into convenient form for relating the total and partial molal enthalpy and free-energy functions in the form in which they are expressed in this paper, as well as immediately giving the standard heat and free energy of formation of ZrH*_x_* at temperature *T.* In these two cases eq (5) becomes respectively

$$H{}^{\circ}={\bar{H}}_{1}+\frac{1}{2}x{\bar{H}}_{2}$$ (37)

and

$$G{}^{\circ}={\bar{G}}_{1}+\frac{1}{2}x{\bar{G}}_{2}.$$ (38)

Subtracting eq (37) from eq (32) (and remembering eq (13)) gives

$$\Delta Hf{}^{\circ}/T=\left({\bar{H}}_{1}-H_{1}^{{}^{\circ}}\right)/T+\frac{1}{2}x\left({\bar{H}}_{2}-H_{2}^{{}^{\circ}}\right)/T=\left(H{}^{\circ}-H_{298}^{{}^{\circ}\left(\alpha +\delta \right)}\right)/T-\left(H_{1}^{{}^{\circ}}-H_{1\left(298\right)}\right)/T-\frac{1}{2}x\left(H_{2}^{{}^{\circ}}-H_{2\left(298\right)}^{{}^{\circ}}\right)/T+{\left({\bar{H}}_{1}^{\alpha +\delta }+H_{1}^{{}^{\circ}}\right)}_{298}/T+\frac{1}{2}x{\left({\bar{H}}_{2}^{\alpha +\delta }+H_{2}^{{}^{\circ}}\right)}_{298}/T,$$ (39)

whereas subtracting eq (38) from eq (32) (and remembering eq (15)) gives

$$\Delta Gf{}^{\circ}/T=\left({\bar{G}}_{1}-G_{1}^{{}^{\circ}}\right)/T+\frac{1}{2}x\left({\bar{G}}_{2}-G_{2}^{{}^{\circ}}\right)/T=\left(G{}^{\circ}-H_{298}^{{}^{\circ}\left(\alpha +\delta \right)}\right)/T-\left(G_{1}^{{}^{\circ}}-H_{1\left(298\right)}^{{}^{\circ}}\right)/T-\frac{1}{2}x\left(G_{2}^{{}^{\circ}}-H_{2\left(298\right)}^{{}^{\circ}}\right)/T+{\left({\bar{H}}_{1}^{\alpha +\delta }+H_{1}^{{}^{\circ}}\right)}_{298}/T+\frac{1}{2}x{\left({\bar{H}}_{2}^{\alpha +\delta }+H_{2}^{{}^{\circ}}\right)}_{298}/T.$$ (40)

## 4. Alpha-Zirconium

Pure metallic zirconium undergoes a first-order transformation from the hexagonal (*α*) to the high-temperature body-centered-cubic (*β*) lattice. The transformation temperature has been given values from 1,135 °K [21] to 1,143 °K [22, 23]. The value adopted in this paper is:

TransformationtemperatureofZr=1,136°K. (41)

Measurements of the low-temperature heat capacity of zirconium have been reported by Todd (53 to 297 °K) [24]; Skinner and Johnston (14 to 298 °K) [25]; Estermann, Friedberg, and Goldman (1.8 to 4.2 °K) [26]; Wolcott (1.2 to 20 °K) [27]; and Burk, Estermann, and Friedberg (20 to 200 °K) [28]. The heat capacities of Todd and those of Burk, Estermann, and Friedberg are in close agreement, while those obtained above 130 °K by Skinner and Johnston are about 1 percent lower. The enthalpy at high temperatures has been measured by Mixter and Dana (273 to 373 °K) [29]; Jaeger and Veenstra (294 to 1,074 °K) [30]; Coughlin and King (298 to 1,371 °K) [31]; Redmond and Lones (273 to 1,309 °K) [32]; and Douglas and Victor (273 to 1,173 °K) [16]. Furukawa and Reilly [33] have carefully analyzed the low-temperature data and have derived a table of thermodynamic functions from 0 to 300 °K whose heat capacities approach those of Douglas and Victor at and above 300 °K. The heat-capacity curve of Douglas and Victor joins smoothly with that of Todd, but not with that of Skinner and Johnston. At 250 °K Furukawa and Reilly’s table gives (in the notation of sec. 2)

$${\left(C_{p}^{{}^{\circ}}\right)}_{1\left(250\right)}=5.969$$ (42)

and

$${\left(S{}^{\circ}\right)}_{1\left(250\right)}=8.219.$$ (43)

Using a sample of zirconium containing a total of 0.09 percent by weight of impurities (0.015% Hf, 0.03% Fe, 0.02% C, 0.005% N, and 0.006% O), Douglas and Victor [16] precisely measured the enthalpy, relative to 273 °K, at 100-deg intervals up to 1,073 °K, and also at 1,123° and at 5-deg intervals from 1,143 to 1,173 °K. The following equation (in the notation of sec. 2) was derived to fit their data for the *α* form and to satisfy eq (42):

$$H_{1}^{{}^{\circ}}-H_{1\left(298.15\right)}^{{}^{\circ}}=4.0504T+5.3113\left(10^{-3}\right)T^{2}-4.4945\left(10^{-6}\right)T^{3}+1.6785\left(10^{-9}\right)T^{4}-1573.9.$$ (44)

The mean observed values differ from this equation by the amounts shown in table 2.

For the entropy of *α*-zirconium, eq (43) and (44) give

$$S_{1}^{{}^{\circ}}=9.32645\;\log\;T+1.06226\left(10^{-2}\right)T-6.74175\left(10^{-6}\right)T^{2}+2.238\left(10^{-9}\right)T^{3}-16.4145.$$ (45)

The thermodynamic properties of *β*-zirconium will be evaluated in section 7 in connection with the *β* field of the Zr-H system, of which it forms a part.

## 5. The *α* and (*α*+*β*) Phase Fields

The thermodynamic properties of the *α* and (*α*+*δ*) phase fields as derived in this paper are closely related, and will be discussed together. Other parts of the Zr-H system will be similarly treated in the following two sections.

### 5.1. Choice of the *α*-Phase Eutectoid Point

Values for the eutectoid temperature, where the *α*, *β*, and *δ* phases of fixed compositions coexist in equilibrium, are in fair agreement as reported by various investigators. Vaughan and Bridge [10] gave 833°±10 °K, but the values most commonly reported have been closer to that of Ells and McQuiilan [6], which is 820°±2 °K. In an enthalpy investigation [16], four samples transformed somewhere between 823 and 833 °K, and one sample somewhere between 813 and 823 °K. The exact temperature is probably uncertain by a few degrees owing to some sluggishness in the (all solid) transformation, but for the purposes of the calculations of this paper the following value has been adopted:

Eutectoidtemperature=820°K. (46)

Another constant evaluated empirically here is the eutectoid composition of the *α* phase. As indicated in figure 1, the *α*/(*α*+*δ*) and *α*/(*α*+*β*) boundaries of different investigators [4,6,7,8,9] are in fairly good agreement. On the basis of these results the composition at 820°K selected here is

$$x_{\alpha }\left(\text{eutectoid}\right)=0.0650.$$ (47)

The equilibrium properties of the (*α*+*δ*) field are of course the appropriate combinations of the properties of the component phases along the two phase-field boundaries, which if known can be used to derive the two-phase-field properties. This procedure has been followed for the (*α*+*β*) field (sec. 7). If, however, more complete data are available in the two-phase field, these may be used to derive its properties even without knowing the boundaries of the field. In the present case some hydrogen-pressure data are available for the higher temperatures of the field, but not at the lower temperatures near room temperature, mainly because the equilibrium pressures are negligibly small.

### 5.2. Enthalpy and Related Properties

The enthalpy measurements on five well-spaced compositions in the (*α*+*δ*) field [16,17] show good precision and good consistency, and will be used as the basis for extending other properties below the eutectoid temperature. These data show not only the linearity with *x* of isotherms expected for a two-phase field [16,17], but show also other evidence, soon to be presented, that phase equilibrium was approximately maintained during the rather rapid cooling involved in the measurements.^8^ Representing an isotherm of enthalpy by

$${\left(H{}^{\circ}-H_{298.15}^{{}^{\circ}}\right)}^{\alpha +\delta }=A+Bx,$$ (48)

the values of *A* and *B* (referred to the experimental base temperature of 273.15 °K) were determined from the data by the least-square method, and are given in table 3 referred to the experimental base temperature 273.15 °K—i.e., for the equation

$${\left(H{}^{\circ}-H_{273.15}^{{}^{\circ}}\right)}^{\alpha +\delta }=A^{\prime }+B^{\prime }x.$$ (49)

The following equations were derived to represent *A* and *B* in eq (48) as functions of temperature *(T*=273 to 823)^9^:

$$A=33.91211T-5.051385\left(10^{-}{\;}^{2}\right)T^{2}+4.76757\left(10^{-5}\right)T^{3}-1.603496\left(10^{-8}\right)T^{4}+1.373028\left(10^{6}\right)T^{-1}-9.2289104\left(10^{7}\right)T^{-2}-10324.4;$$ (50)

$$B=3.05464T+5.5805\left(10^{-4}\right)T^{2}-7.098895\left(10^{-5}\right)T^{3}+2.608868\left(10^{-7}\right)T^{4}-3.39327\left(10^{-10}\right)T^{5}+1.520416\left(10^{-13}\right)T^{6}-447.8.$$ (51)

From eqs (50) and (51), the constants in eqs (48) and (49) are related by

$$A^{\prime }=A+158.1;$$ (52)

$$B^{\prime }=B+19.0.$$ (53)

From eqs (1), (6), (8), and (48)

$${\left({\bar{C}}_{p}\right)}_{1}{\;}^{\alpha +\delta }=dA/dT$$ (54)

and

$${\left({\bar{C}}_{p}\right)}_{2}{\;}^{\alpha +\delta }=2dB/dT.$$ (55)

From eqs (26), (28), (29), (48), and (51)

$$\left({\bar{H}}_{2}{\;}^{\alpha +\delta }-H_{2}^{{}^{\circ}}\right)/T=-40404-0.55072T+8.483\left(10^{-4}\right)T^{2}-1.419779\left(10^{-4}\right)T^{3}+5.217736\left(10^{-7}\right)T^{4}-6.78654\left(10^{-10}\right)T^{5}+3.040832\left(10^{-13}\right)T^{6}+12000T^{-1}.$$ (56)

The same properties were next formulated in the *α* field. Apparently no measurements of enthalpy are available except for zirconium metal (sec. 4). However, the *α* phases are all low in hydrogen (fig. 1), and the laws of dilute solutions should be applicable to good approximation. This involves the assumption that the enthalpy and heat-capacity isotherms are linear with *x.* It will presently be shown that at 273.15 °K the *α* field is extremely narrow; consequently, it is convenient to assume that at this temperature a-Zr is in the (*α*+*δ*) field. At 820 °K eq (44) then gives

$${\left(H_{820}^{{}^{\circ}\left(\alpha \right)}-H_{273.15}^{{}^{\circ}\left(\alpha +\delta \right)}\right)}_{x=0}=3753.0,$$ (57)

while eqs (47), (48), (50), (51), (52), and (53) give

$${\left(H_{820}^{{}^{\circ}\left(\alpha \right)}-H_{273.15}^{{}^{\circ}\left(\alpha +\delta \right)}\right)}_{0.0650}=4359.8.$$ (58)

Thus, using eqs (52) and (53),

$$H_{820}^{{}^{\circ}\left(\alpha \right)}-H_{298.15}^{{}^{\circ}\left(\alpha +\delta \right)}=3594.9+9316.4x.$$ (59)

By assuming the hydrogen vibration frequency in ZrH_2_ found by Flotow and Osborne [15], 1190 cm^−1^, the following empirical equations were derived:

$$T=298-820:{\left({\bar{C}}_{p}\right)}_{2}^{\alpha }=-5.9446+2.7364\left(10^{-2}\right)T-1.185\left(10^{-5}\right)T^{2}$$ (60)

$$T=820-1136:{\left({\bar{C}}_{p}\right)}_{2}^{\alpha }=4.836+4.5\left(10^{-3}\right)T$$ (61)

Since these are independent of *x*, by eqs (9) and (8) ${\left({\bar{C}}_{p}\right)}_{1}^{\alpha }$ is identical with the heat capacity of a-Zr (from eq (44)). When the latter and eq (60) (or (61)) are substituted into eq (5) and then integrated to satisfy eq (59), we have:

$$T=298-820:\left(H^{{}^{\circ}\left(\alpha \right)}-H_{298.15}^{{}^{\circ}\left(\alpha +\delta \right)}\right)/T=4.0504+5.3113\left(10^{-3}\right)T-4.4945\left(10^{-6}\right)T^{2}+1.6785\left(10^{-9}\right)T^{3}-1578.5/T-\left[8243.0/T-2.9723+6.841\left(10^{-3}\right)T-1.975\left(10^{-6}\right)T^{2}\right]x;$$ (62)

$$T=820-1136:\left(H^{{}^{\circ}\left(\alpha \right)}-H_{298.15}^{{}^{\circ}\left(\alpha +\delta \right)}\right)/T=4.0504+5.3113\left(10^{-3}\right)T-4.4945\left(10^{-6}\right)T^{2}+1.6785\left(10^{-9}\right)T^{3}-1578.5/T+\left[6577.4/T-2.418+1.125\left(10^{-3}\right)T\right]x.$$ (63)

The application of eqs (26), (28), and (29) to eqs (62) or (63) gives respectively:

$$T=298-820:\left({\bar{H}}_{2}^{\alpha }-H_{2}^{{}^{\circ}}\right)/T=-23005/T-12.6085+1.3414\left(10^{-2}\right)T-3.950\left(10^{-6}\right)T^{2};$$ (64)

$$T=820-1136:\left({\bar{H}}_{2}^{\alpha }-H_{2}^{{}^{\circ}}\right)/T=-26336/T-1.828+1.982\left(10^{-3}\right)T.$$ (65)

### 5.3. Hydrogen Activity and the *α*/(*α*+*δ*) Boundary

The thermodynamic functions $\left({\bar{G}}_{2}-G_{2}^{{}^{\circ}}\right)/T$, giving (by eq (10)) the hydrogen equilibrium pressures, will now be formulated for the *α* and (*α*+*δ*) fields. As discussed in section 3, these will be based on eq (23) and the pertinent thermal properties. The invariant eutectoid pressure derived in section 6 is the *(α*+*δ*) value at that temperature:

$$a_{2\left(820\right)}^{\alpha +\delta }=1.0442\left(10^{-4}\right).$$ (66)

It is reasonable to assume that in the dilute *α* phases the activity of atomic hydrogen is proportional to its concentration at any temperature, and hence $a_{2}^{\alpha }$ is proportional to *x*^2^, as has been observed [4]. Then from eqs (10) and (66)

$${\left[\left({\bar{G}}_{2}^{\alpha +\delta }-G_{2}^{{}^{\circ}}\right)/T\right]}_{820}=-18.2174,$$ (67)

and, using eq (47),

$${\left[\left({\bar{G}}_{2}^{\alpha }-G_{2}^{{}^{\circ}}\right)/T\right]}_{820}=-9.1517\;\log\;x-7.3536.$$ (68)

Substitution of eq (56) into eq (11) and integrating to satisfy eq (67),

$$\left({\bar{G}}_{2}^{\alpha +\delta }-G_{2}^{{}^{\circ}}\right)/T=-40,403.48/T+1.26796\;\log\;T-8.4829\left(10^{-4}\right)T+6000/T^{2}+7.098842\left(10^{-5}\right)T^{2}-1.739232\left(10^{-7}\right)T^{3}+1.696622\left(10^{-10}T^{4}\right)-6.08162\left(10^{-14}\right)T^{5}+22.0491.$$ (69)

The similar use of eqs (11), (64), and (68) gives

$$T=298-820:\left({\bar{G}}_{2}^{\alpha }-G_{2}^{{}^{\circ}}\right)/T=9.1517\;\log\;x-23005/T+29.02815\;\log\;T-1.34113\left(10^{-2}\right)T+1.97447\left(10^{-6}\right)T^{2}-54.116,$$ (70)

while eqs (11), (65), and (68) give

$$T=820-1136:\left({\bar{G}}_{2}^{\alpha }-G_{2}^{{}^{\circ}}\right)/T=9.1517\;\log\;x-26336/T+4.20976\;\log\;T-1.98179\left(10^{-3}\right)T+14.1221.$$ (71)

The *α/(α*+*δ*) boundary at 820 °K has been required to have a composition of *x_α_*= 0.0650, but no other restrictions on it have been explicitly imposed. The boundary compositions at other temperatures can be found by equating either the hydrogen activities (eqs (69) and (70)) or the enthalpies (eqs (48), (50), (51), (62)) in the two fields. The results by these two methods are compared in table 4.^10^ The agreement for the two methods above 400 °K is rather striking, and gives strong support to the assumption made earlier that in the *α* field *a*_2_ is proportional to *x*^2^. If this assumption is correct, the agreement in the table indicates that phase equilibrium, especially with respect to the *α*/(*α*+*δ*) boundary, was approximately maintained in the enthalpy measurements in the (*α*+*δ*) field, a result in accord with the observation of Schwartz and Mallett [9] that hydrogen diffuses in zirconium metal very rapidly at these temperatures. Aside from the small uncertainty in the value selected for 820 °K, the values in the second column of table 4 are probably more reliable than those in the third column; this is certainly true at the lowest temperatures, where the failure of the *α* phase to reach the final equilibrium compositions would have caused but little error in eq (56) and hence in eq (69). In this paper, however, the properties of the *(α*+*δ*) field depending on the *α/(α*+*δ*) boundary are calculated from the third column of the table in order to avoid small discrepancies with other properties calculated from the thermal data. These discrepancies would have been minor, because the two columns of table 4 never differ by more than about 0.001 in *x.* For the same reason, no effort was made to adjust the *(α*+*δ*) enthalpy values to make them consistent with the second-column boundary compositions.

### 5.4. Entropy

The absolute entropies of the various hydride compositions are calculated in this paper from those of zirconium metal (eq (45)). $\bar{S_{2}^{\alpha }}$ can bo calculated from eq (4) by substituting either eqs (64) and (70) or eqs (65) and (71), depending on the temperature. Specifically, at 820 °K $S_{2}^{{}^{\circ}}=38.285$, and so we get

$${\bar{S}}_{2\left(820\right)}^{\alpha }=13.3187-9.1516\;\log\;x.$$ (72)

Since from eq (45)

$$S_{1\left(820\right)}^{{}^{\circ}}=16.1723,$$ (73)

eqs (72) and (73) may be substituted into eq (7) and the integration performed from *x*=0 to *x*, giving

$$S_{\left(820\right)}^{{}^{\circ}\left(\alpha \right)}=16.1723+8.6466x-4.5758x\;\log\;x.$$ (74)

The entropy may be formulated from the enthalpy by performing the integration (of eq (2)) to satisfy eq (74). When eq (63) is used for the enthalpy, the result is

$$T=820-1136:S{{}^{\circ}}^{\left(\alpha \right)}=\left(9.32645+5.5677x\right)\;\log\;T+\left[0.0106226+2.25\left(10^{-3}\right)x\right]T-6.74175\left(10^{-6}\right)T^{2}+2.238\left(10^{-9}\right)T^{3}-16.4145-9.4216x\;\log\;x.$$ (75)

Equation (74) gives (in view of eqs (46) and (47))

$$S_{820,0.0650}^{0\left(\alpha \right)}=S_{820,0.0650}^{0\left(\alpha +\delta \right)}=17.874.$$ (76)

At this temperature eqs (4), (56), and (69) and the value of ${\left(S_{2}^{{}^{\circ}}\right)}_{820}$ give

$${\bar{S}}_{2\left(820\right)}^{\alpha +\delta }=5.5207.$$ (77)

The substitution from eqs (76) and (77) into eq (5) then gives

$${\bar{S}}_{1\left(820\right)}^{\alpha +\delta }=16.9080.$$ (78)

${\left({\bar{C}}_{p}\right)}_{1}^{\alpha +\delta }$ and ${\left({\bar{C}}_{p}\right)}_{2}^{\alpha +\delta }$ as functions of temperature were then evaluated from eqs (50, 54) and (51, 55) respectively, and when each of these functions was substituted into the equation for the partial molal properties analogous to eq (2) and the subsequent integration was required to satisfy eq (78) or (77) respectively, the partial molal and (from eq (5)) the total entropies in the (*α*+*δ*) field were completely determined. The results for the partial molal entropies are

$${\bar{S}}_{1}^{\alpha +\delta }=78.08602\;\log\;T-0.1010277T+7.151355\left(10^{-5}\right)T^{2}-2.13799467\left(10^{-8}\right)T^{3}+6.86514\left(10^{5}\right)T^{-2}-6.15260693\left(10^{7}\right)T^{-3}-164.9841;$$ (79)

$${\bar{S}}_{2}^{\alpha +\delta }=14.06722\;\log\;T+2.2322\left(10^{-3}\right)T-2.1296685\left(10^{-4}\right)T^{2}-6.95698132\left(10^{-7}\right)T^{3}-8.483175\left(10^{-10}\right)T^{4}+3.6489984\left(10^{-13}\right)T^{5}-29.4258.$$ (80)

### 5.5. Some Partial Molal Properties With Respect to Zirconium

In the *α* field the partial molal relative free energy and relative enthalpy of zirconium may be found readily from those for hydrogen by integrating the Gibbs-Duhem equation (eq (9)), since zirconium in its standard state is a terminal composition (*x *= 0) of this field. Equations (70) and (71) both give the same result for the relative free energy,

$$\left({\bar{G}}_{1}^{\alpha }-G_{1}^{0}\right)/T=-1.9872x,$$ (81)

and eqs (64) and (65) give the same result for the relative enthalpy,

$$\left({\bar{H}}_{1}^{\alpha }-H_{1}^{0}\right)/T=0.$$ (82)

^11^In the *(α*+*δ*) field the partial molal relative free energy of zirconium is simply the values given by eq (81) along the *α/(α*+*δ*) boundary:

$$\left({\bar{G}}_{1}^{\alpha +\delta }-G_{1}^{0}\right)/T=-1.9872x_{a}.$$ (83)

The values of *x_α_* are given in table 4, where, as explained earlier, those in the third column were used to obtain the tabulated values of $\left({\bar{G}}_{1}^{\alpha +\delta }-G_{1}^{0}\right)/T$.

The partial molal relative enthalpy of zirconium in the (*α*+*δ*) field may be obtained from eq (4), which in this case is

$$\left({\bar{H}}_{1}^{\alpha +\delta }-H_{1}^{0}\right)/T=\left({\bar{G}}_{1}^{\alpha +\delta }-G_{1}^{0}\right)/T+{\bar{S}}_{1}^{\alpha +\delta }-S_{1}^{0},$$ (84)

by substituting from eqs (45), (79), and (83). For the zirconium-hydrogen system this property may be derived more directly from the enthalpies alone by what is equivalent to the “method of intercepts.” From eq (8)

$${\bar{H}}_{1}=H^{0}-x\partial H^{0}/\partial x.$$ (85)

By writing the same equation for 273 °K and the (*α*+*δ*) field and subtracting from eq (85), we may write

$$\left({\bar{H}}_{1}-H_{1}^{0}\right)=\left({\bar{H}}^{0}-H_{273}^{0\left(\alpha +\delta \right)}\right)-x{\left[\frac{\partial }{\partial x}\left(H^{0}-H_{273}^{0\left(\alpha +\delta \right)}\right)\right]}_{T}-\left(H_{1}^{0}-H_{1\left(273\right)}^{0}\right)+{\left({\bar{H}}_{1}^{\alpha +\delta }+H_{1}^{0}\right)}_{273}.$$ (86)

It was stated earlier, however, that *α*-Zr will be assumed to lie in the (*α*+*δ*) field at 273 °K so that

$${\left({\bar{H}}_{1}^{\alpha +\delta }-H_{1}^{0}\right)}_{273}=0.$$ (87)

From eqs (48), (49), (52), and (53),

$$\left(H_{298}^{0\left(\alpha +\delta \right)}-H_{273}^{0\left(\alpha +\delta \right)}\right)-x{\left[\frac{\partial }{\partial x}\left(H_{298}^{0\left(\alpha +\delta \right)}-H_{273}^{0\left(\alpha +\delta \right)}\right)\right]}_{T}=158,$$ (88)

and from eq (44),

$$H_{1\left(298\right)}^{0}-H_{1\left(273\right)}^{0}=154.$$ (89)

Subtracting eq (89) from the sum of eqs (86), (87), and (88),

$$\left({\bar{H}}_{1}-H_{1}^{0}\right)/T=\frac{1}{T}\left\{\left(H^{0}-H_{298}^{0\left(\alpha +\delta \right)}\right)-x{\left[\frac{\partial \left(H^{0}-H_{298}^{0\left(\alpha +\delta \right)}\right)}{\partial x}\right]}_{T}-\left(H_{1}^{0}-H_{1\left(298\right)}^{0}\right)+4\right\}.$$ (90)

For the (*α*+*δ*) field at *T*= 298, eq (90) gives the value of eq (25). Equation (90) is applicable to any phase field and any composition lying inside the *(α*+*δ*) field at 298 °K. In the special case of the (*α*+*δ*) field, substitution from eq (48) gives the simple result:

$$\left({\bar{H}}_{1}^{\alpha +\delta }-H_{1}^{0}\right)/T=\left[A-\left(H_{1}^{0}-H_{1\left(298\right)}^{0}\right)+4\right]/T.$$ (91)

Values calculated from eqs (84) and (91) did not differ by more than 0.006, and were averaged for tabulation.

## 6. The *β* (Hydrogen-Rich), (*β*+*δ*) and *δ* Phase Fields

Having formulated thermodynamic properties for the *(α*+*δ*) field, those formulated for the *β*, *β*+*δ*, and *δ* fields should be consistent not only with the most reliable data for these fields but also with a single set of *β*/(*β*+*δ*), *δ*/(*β*+*δ*), and *δ*/(*α*+*δ*) boundaries. Therefore the properties of these three fields are considered together.

An isotherm of *a*_2_ at 1073.2 °K for the *β* field has already been formulated in section 3 (eq (23)). As the hydrogen concentration *(x*) increases, the second term in this equation becomes relatively important (several percent of the first term) in the region *x*= 0.5 to 0.6; other properties such as the heat capacity and the heat of hydriding $\left({\bar{H}}_{2}-H_{2}^{0}\right)$, at similar temperatures and the same general range of concentrations, show similar changes. This fact makes it convenient to treat the wide *β* field separately in two parts. Somewhat arbitrarily, the field has been divided in this paper by the composition selected as that of the *β* eutectoid phase, *x*=0.57. The properties of the *β* phases richer in hydrogen will be considered quantitatively in the present section, and those poorer in hydrogen, in section 7. The most appropriate dividing composition probably shifts with temperature, but the exact choice is probably not of major importance because all the important thermodynamic properties are made to have continuity at this composition.

### 6.1. The *β* Enthalpy and Other Properties (*x*≧0.57)

A simple equation was selected to fit the enthalpy measurements [16] in the *β* field for the higher values of *x*:

$$x≧0.57:\left(H^{0\left(\beta \right)}-H_{298.15}^{0\left(\alpha +\delta \right)}\right)/T=\left(9.664+2.144x\right)T+1500x-2521.1.$$ (92)

The values calculated from this equation are compared in table 5 with the mean observed enthalpies.^12^ (The latter were actually measured relative to 273.15 °K, but have been made relative to 298.15 °K by subtracting the quantities indicated by eqs (52) and (53).)

If any of the samples was actually inside the (*β*+*δ*) field when measured, its enthalpy would be found to be lower than if at the same temperature it were in the *β* field. Measurements were made on the compositions *x*=0.556 and 0.701 also at 873.2 °K, but, contrary to figure 1, these enthalpies gave definite evidence (poor precision, large thermal hysteresis, and large negative deviations from eq (92)) that for some reason the *β* field had not been reached. By similar considerations the observed values in table 5 for *x*=0.999 at *T*=1123.2 and for *x*=1.071 at *T*=1123.2 and 1073.2 would be expected to fall below eq (92). The fact that the last value is only slightly lower is evidence that the preceding value (for *x*=1.071 and *T*= 1123.2) probably corresponds to the *β* field, despite the *β*/(*β*+*δ*) boundary selected in figure 1. Most likely, small percentages of impurities in each sample, while corrected for additively, probably invalidated the observed enthalpy appreciably only if the impurities shifted this phase-field boundary enough to place the point erroneously in a two-phase field. The doubtful values mentioned were not used in deriving eq (92). It may be noted from table 5 that the fit is poorer and with a trend for the two lowest temperatures tabulated (up to 0.5% discrepancy), but these four points were considered insufficient justification for modifying eq (92).

With the help of eqs (28), (29), and (92), eq (26) gives:

$$x≧0.57:\left({\bar{H}}_{2}^{\beta }-H_{2}^{0}\right)/T=-36508.3/T-2.372-2.678\left(10^{-4}\right)T+12000/T^{2}.$$ (93)

When eq (93) is substituted into eq (11), and the subsequent integration is made to satisfy eq (24), there is obtained:

$$x≧0.570:\left({\bar{G}}_{2}^{\beta }-G_{2}^{0}\right)/T=6.2231\;\log\;x+4.5758\;\log\;\left(1+1.8493x^{6.97}\right)+11.6100-36508.3/T+5.4617\;\log\;T+2.677\left(10^{-4}\right)T+6000/T^{2}.$$ (94)

Though eq (24) was derived from data up to about *x*=1, where the *β* field terminates at 1073 °K, eq (94) is assumed to hold up to 1200 °K and also for the slightly greater values of *x* (up to about 1.1) which lie in the *β* field above 1073 °K. An empirical equation was derived for the standard entropy of hydrogen gas from eq (28) (using eqs (1) and (2)). Since eq (28) is not quite exact, the integrated equation was adjusted to give the best fit for *T*≧820:

$$T≧820:S_{2}^{0}=15.33532\;\log\;T+5.363\left(10^{-4}\right)T-6000/T^{2}-6.8288.$$ (95)

Substitution from eqs (93), (94), and (95) into eq (4) then gives:

$$x≧0.57:{\bar{S}}_{2}^{\beta }=-6.2231\;\log\;x-4.5758\;\log\;\left(1+1.8493x^{6.97}\right)+9.8736\;\log\;T-20.8108.$$ (96)

Substituting from eqs (44) and (92) into eq (90),

$$x≧0.57:\left({\bar{H}}_{1}^{\beta }-H_{1}^{0}\right)/T=-942.6/T+5.6136-5.3113\left(10^{-3}\right)T+4.4945\left(10^{-6}\right)T^{2}-1.6785\left(10^{-9}\right)T^{3}.$$ (97)

Other properties to be derived for the hydrogen-rich part of the *β* field will depend on how the (*β*+*δ*) properties are formulated, because these, together with the *β* properties, will determine the unique composition of the *β* eutectoid phase with which they are consistent.

### 6.2. Procedure Giving Thermodynamic Consistency

Of the twelve thermodynamic functions derived and tabulated in this paper (tables 8–14), five must be continuous at each phase-field boundary: *H, S, G*, ${\bar{G}}_{1}$, and ${\bar{G}}_{2}$. Both relative-enthalpy and hydrogen-activity data have been obtained in the (*β*+*δ*) field. The enthalpy data were obtained by Douglas and Victor [16] for the compositions *x*=0.701, 0.999, and 1.071, but the values show serious inconsistencies between two of the sample compositions,^14^ and therefore were given no weight in deriving properties in this field. Hydrogen-activity values in the (*β*+*δ*) field were reported as follows: by Edwards et al. [5] for *x*=1.222 at 973, 1,023, 1,073, 1,098, 1,123, 1,133, 1,138, and 1,148 °K; by LaGrange et al. [8] for several compositions at 843, 889, 919, 967, 1,022, and 1,073 °K. The values of the two sets of workers, which disagree somewhat at the lower temperatures, show excellent agreement as well as smooth temperature dependence at and above 1,023 °K, and were used to define the adopted values in this higher temperature range. With the complete formulation of the hydrogen activity (or ${\bar{G}}_{2}{\;}^{\beta +\delta }$) from the eutectoid temperature (820 °K) to 1,200 °K, soon to be given, the *β*/(*β*+*δ*) boundary was so determined as to make ${\bar{G}}_{2}^{\beta }$ and ${\bar{G}}_{2}^{\beta +\delta }$ equal along the boundary, and the values of the other four of the above five properties in the (*β*+*δ*) field were determined so that along the boundary they would be equal at each temperature to the corresponding *β*-field properties.

Representing the boundary composition at temperature *T* by *x_β_*, the relative enthalpy in the (*β*+*δ*) field at *x* and *T* can be found by integrating eq (26) between *x* and *x_β_*:

$$\left(H{{}^{\circ}}^{\left(\beta +\delta \right)}-H_{298.15}^{{}^{\circ}\left(\alpha +\delta \right)}\right)/T={\left(H{{}^{\circ}}^{\left(\beta \right)}-H_{298.15}^{{}^{\circ}\left(\alpha +\delta \right)}\right)}_{x_{\beta }}/T+\left[{\bar{H}}_{2}^{\beta +\delta }-H_{2}^{{}^{\circ}})-{{\bar{H}}_{2}^{\alpha +\delta }-H_{2}^{{}^{\circ}})}_{298}+\left(H_{2}^{{}^{\circ}}-H_{2\left(298\right)}^{{}^{\circ}}\right)\right]\left[\left(x-x_{\beta }\right)/2T\right].$$ (98)

The complete determination of $\left({\bar{H}}_{2}^{\beta +\delta }-H_{2}^{{}^{\circ}}\right)$ at the lower temperatures down to 820 °K will then complete the determination of the (*β*+*δ*) properties and the *β*/(*β*+*δ*) boundary. In particular, the composition of the eutectoid *β* phase will lie on this boundary, and the composition of the eutectoid 5 phase will be that resulting from simultaneous solution of eqs (48) and (98) at 820 °K, the eutectoid temperature. It was decided to formulate thermodynamic properties of the Zr–H system only up to *x*=1.25, partly because there are fewer overlapping data for compositions richer in hydrogen and those that do exist show some major inconsistencies. If the eutectoid 5 phase has a value of *x* less than 1.25, then as the temperature rises the composition *x*=1.25 passes from the (*α*+*δ*) field into the 5 field at some temperature (*T*_1_, and from the 5 into the (*β*+*δ*) field at a higher temperature (*T*_2_). For thermodynamic consistency, it must be possible to formulate the *δ*-phase properties so that the five properties mentioned above agree with those of the *(α*+*δ*) and (*β*+*δ*) fields at the eutectoid *δ* composition and temperature (820 °K), with the *(α*+*δ*) properties at *x*=1.25 and *T*_1_, and with the (*β*+*δ*) properties at *x*=1.25 and *T*_2_.

The (*β*+*δ*) hydrogen-activity data above 1073 °K mentioned above indicate a value of $\left({\bar{H}}_{2}^{\beta +\delta }-H_{2}^{{}^{\circ}}\right)$ essentially independent of temperature (approximately −51600), and in a preliminary trial the same value was assumed down to the eutectoid temperature. This assumption led, in the manner outlined above, to eutectoid compositions of approximately *x_β_* = 0.54 and *x_β_*=0.99. The next step taken was to formulate an enthalpy function for the *δ* phases whose temperature derivative corresponded to a hydrogen vibration frequency of between 1050 and 1200 cm^−1^ [15] and which at the same time satisfied the (*α*+*δ*) enthalpy equation (eq (48)) at the eutectoid *δ* point and also on the *δ*/(*α*+*δ*) boundary at 673 °K. With *x*=1.25, this equation was then solved simultaneously with eq (48) to determine *T*_1_ and with eq (98) to determine *T*_2_. To test the continuity of the other four properties at the points *T*_1_ and *T*_2_, entropy is an insensitive criterion, but ${\bar{G}}_{2}$ proves entirely adequate. The *δ*-phase enthalpy function mentioned above and eq (26) gave a value of approximately $\left({\bar{H}}_{2}^{\delta }-H_{2}^{\delta }\right)=-42900$, and on substituting this value, eq (11) integrated to give for ${\left[\left({\bar{G}}_{2}^{\delta }-G_{2}^{{}^{\circ}}\right)/T\right]}_{T_{2},1.25}-{\left[\left({\bar{G}}_{2}^{\delta }-G_{2}^{{}^{\circ}}\right)/T\right]}_{T_{1},1.25}$ about 0.7 less than ${\left[\left({\bar{G}}_{2}^{\beta +\delta }-G_{2}^{{}^{\circ}}\right)/T\right]}_{T_{2},1.25}-{\left[\left({\bar{G}}_{2}^{\alpha +\delta }-G_{2}^{{}^{\circ}}\right)/T\right]}_{T_{1},1.25}$. (The discrepancy of 0.7 was approximately 15% of either of the two differences.)

This serious discrepancy was practically eliminated by making two changes. The first change was to preserve the fit to the hydrogen-activity data at the higher temperatures but to assume slightly higher values of $\left({\bar{H}}^{\beta +\delta }-H_{2}^{{}^{\circ}}\right)$ at the lowest temperatures (near 820 °K). The final equations are

$$\left({\bar{H}}_{2}^{\beta +\delta }-H_{2}^{{}^{\circ}}\right)/T=\left[-51678.2+3.1277(10^{-10}){\left(1200-T\right)}^{5}\right]/T$$ (99)

and (from eq (11) after substituting from eq (99))

$$\left({\bar{G}}_{2}^{\beta +\delta }-G_{2}^{{}^{\circ}}\right)/T=726592\;.5/T+\;7466.81126\;\log\;T-5.4046582\;T\;+\;2\;.2519409\;(10^{-3})\;T^{2}-6.2553915\;(10^{-7})\;T^{3}+7\;.81924(10^{-11})\;T^{4}-19434.0399.$$ (100)

The additional eutectoid compositions (at 820 °K) were then found in the ways outlined above. Simultaneous solution of eqs (94) and (100) gave

$$x_{\beta }\left(\text{eutectoid}\right)=0.570.$$ (101)

Using this value, simultaneous solution of eqs (48) and (98) (after substitution from eqs (50) and (51) into the former, and from eqs (28), (29), (92), and (99) into the latter) gave

$$x_{\delta }\left(\text{eutectoid}\right)=1.171.$$ (102)

The effect of increasing the value of *x_δ_* (eutectoid) from 0.99 to 1.171 was to bring the boundary temperatures *T*_1_ and *T*_2_ (at *x*=1.25) much closer together with the result that the discrepancy in the interval of ${\left({\bar{G}}_{2}^{\delta }-G_{2}^{{}^{\circ}}\right)}_{x=1.25}$ referred to above was now greatly reduced, but still had the same sign, and a magnitude of 0.14. To repeat the above change on an increased scale and thus to increase the eutectoid composition *x_β_* still further would have increased the discrepancies of the *β/(α*+*β*) boundary (sec. 7) but apparently without eliminating the present discrepancy in ${\left({\bar{G}}_{2}^{\delta }-G_{2}^{{}^{\circ}}\right)}_{1.25}$. This discrepancy was, however, practically eliminated by a second change which, by lowering $\left({\bar{H}}_{2}^{\delta }-H_{2}^{{}^{\circ}}\right)$, makes a small increase in the temperature coefficient of $\left({\bar{G}}_{2}-G_{2}^{{}^{\circ}}\right)/T$ in the *δ* field. This was accomplished by replacing the *δ*-phase enthalpy function by the simple equation

$$x=1.17\;\text{to}\;1.4,\;T=790\;\text{to}\;850:\left(H^{\delta }-H_{298.15}^{\alpha +\delta }\right)/T=13.115-\left(4692.5+19.0x\right)/T.$$ (103)

With the use of eqs (103), (28), and (29), eq (26) now gives

$$x=1.17\;\text{to}\;1.4,\;T=790\;\text{to}\;850:\left({\bar{H}}_{{\;}^{2}}^{\delta }-H_{2}^{{}^{\circ}}\right)/T=-39546.3/T-6.660-2.678\left(10^{-4}\right)T+12000/T^{2}.$$ (104)

Equation (104) gives approximately $\left({\bar{H}}_{2}^{\delta }-H_{2}^{{}^{\circ}}\right)=-45200$, which incidentally is much closer than the preliminary value −42900 to that derived by Gulbransen and Andrew [4] from their data, −45750. The *β*/(*β*+*δ*) boundary on which eqs (94) and (100) give the same values of ${\bar{G}}_{2}$ is that shown in figure 1, and for convenience may be represented (to within ±0.001 in *x*) by the empirical equation

$$T=820\;\text{to}\;1200{}^{\circ}K:x_{\beta }=-1.3995+3.5169\left(10^{-3}\right)\;T-1.2\left(10^{-6}\right)T^{2}+\left[1.19853\left(10^{-15}\right)-2.87092\left(10^{-18}\right)T+1.6655\left(10^{-21}\right)\;T^{2}\right]{\left[1200-T\right]}^{6}.$$ (105)

Equation (101), when substituted into eq (94) (or (100)), gives for the eutectoid activity of hydrogen the value of eq (66) given earlier, 1.0442(10^−4^) atm.^15^

The heat capacity which eq (103) gives corresponds (by eqs (1) and (6)) to

$${\left({\bar{C}}_{p}\right)}_{2}^{\delta }=0,$$ (106)

and this deserves some comment. Though eq (103) was derived to fit only a narrow range of temperatures and compositions, no claim can be made that eq (106) is highly accurate and hence that the addition of hydrogen to the *δ* phases produces an intrinsic addition to the heat capacity by the vibration of the added hydrogen which is exactly offset by a reduction of the intrinsic contribution by the vibration of the zirconium. It seems not unreasonable, however, that a considerable compensation of this kind may actually exist. Equation (80) gives negative values for ${\bar{S}}_{2}^{\alpha +\delta }$ at the lowest temperatures such as 298 °K, which means that the addition of hydrogen to *α*-zirconium to form the coexistent *δ*-phases lowers the entropy at these temperatures, and hence presumably the heat capacities at all lower temperatures. In these cases it is reasonable to assume that a major result of adding the hydrogen has been to stiffen the zirconium lattice, and that this relative effect is dependent on composition as well as on temperature.

### 6.3. Other *β* Properties (*x*≧0.57)

With the value of eq (101), further properties may be derived in the *β* field. From eqs (5), (77), and (78),

$$S_{820,0.570}^{{}^{\circ}\left(\alpha +\delta \right)}=18.4814.$$ (107)

Equations (48), (50), and (51) give

$${\left(H{}^{\circ}-H_{298.15}^{{}^{\circ}}\right)}_{820,0.570}^{\alpha +\delta }=5040.5,$$ (108)

and eq (92) gives

$${\left(H{{}^{\circ}}^{\left(\beta \right)}-H_{298.15}^{{}^{\circ}\left(\alpha +\delta \right)}\right)}_{820,0.570}=7260.5,$$ (109)

whence

$$\Delta S_{820}=2.7073\;\text{for}\;{\mathrm{ZrH}}_{0.570}\left(\alpha +\delta \right)\to {\mathrm{ZrH}}_{0.570}\left(\beta \right).$$ (110)

Adding eqs (107) and (110),

$$S_{820,0.570}^{{}^{\circ}\left(\beta \right)}=21.1887.$$ (111)

For the composition *x*=0.570 eqs (92) and (111) readily give the entropy:

$$S_{0.570}^{{}^{\circ}\left(\beta \right)}=25.06629\;\log\;T-51.8497.$$ (112)

After substituting from eqs (96) and (112), eq (7) can be used to derive the *β* entropies for *x*>0.570. This involves the integral $-2.2879\int_{0.570}^{x}\log\;\left(1+1.8493x^{6.97}\right)\;dx$. Because the expansion of the integral about *x*=0 does not converge rapidly for values of *x* approaching unity, the following empirical equation was derived:

$$\begin{array}{l} x=0.57\;\text{to}\;1.1:-2.2879\int_{0.570}^{x}\log\;\left(1+1.8493x^{6.97}\right)dx\\ \;=0.4202-2.12581x+3.72544x^{2}-2.36774x^{3}+0.18558x^{4}. \end{array}$$ (113)

The general entropy equation then found was

$$x≧0.57:S^{{}^{\circ}\left(\beta \right)}=\left(22.25230+4.93681x\right)\log\;T-46.7017-11.17991x+3.72544x^{2}-2.36774x^{3}+0.18558x^{4}-3.11155x\;\log\;x.$$ (114)

${\bar{S}}_{1}^{\beta }(x≧0.57)$ was calculated from eqs (5), (96), and (114).

Since from eqs (47) and (101) ZrH_0.0650_ (*α*) and ZrH_0.570_(*β*) are coexistent phases at 820 °K, eq (81) (with *x_α_*=0.0650) evaluates ${\bar{G}}_{1}$ for both phases:

$${\left[\left({\bar{G}}_{1}^{\beta }-G_{1}^{{}^{\circ}}\right)/T\right]}_{820,0.570}=-0.1292.$$ (115)

When eq (97) is substituted into eq (11) and the integration is made to satisfy eq (115), there is obtained for the one composition *x*= 0.570

$${\left[{\bar{G}}_{1}^{\beta }-G_{1}^{{}^{\circ}})/T\right]}_{0.570}=-942.6/T-12.92588\;\log\;T+5.3113\left(10^{-3}\right)T-2.24725\left(10^{-6}\right)T^{2}+5.595\left(10^{-10}\right)T^{3}+35.5312.$$ (116)

Equations (94) and (116) may be substituted into the Gibbs-Duhem equation (eq (9)), the integration from *x*=0.570 to higher *x* giving (with the help of eq (113) in integration by parts)

$$x≧0.57:\left({\bar{G}}_{1}^{\beta }-G_{1}^{0}\right)/T=35.9017+0.77449x-3.72544x^{2}+2.36774x^{3}-0.18558x^{4}-2.2879x\;\log\;\left(1+1.8493x^{6.97}\right)-942.6/T-12.92588\;\log\;T+5.3113\left(10^{-3}\right)T-2.24725\left(10^{-6}\right)T^{2}+5.595\left(10^{-10}\right)T^{3}.$$ (117)

### 6.4. Other (*β*+*δ*) Properties

The remaining (*β*+*δ*) properties may be readily derived from foregoing relationships. ${\bar{S}}_{2}^{\beta +\delta }$ may be found from eqs (4), (95), (99), and (100). The entropy was found from that at the *β*/(*β*+*δ*) boundary $(S_{x_{\beta }}^{0\left(\beta \right)}$ calculated from eqs (105) and (114)) and eq (7) in the form

$$S^{0\left(\beta +\delta \right)}=S_{x_{\beta }}^{0\left(\beta \right)}+\frac{1}{2}{\bar{S}}_{2}^{\beta +\delta }\left(x-x_{\beta }\right).$$ (118)

${\bar{S}}_{1}^{\beta +\delta }$ was calculated by substituting *S*^0^*^(β^*^+^*^δ)^* and ${\bar{S}}_{2}^{\beta +\delta }$ into eq (5). $\left({\bar{G}}_{1}^{\beta +\delta }-G_{1}^{0}\right)/T$, which is independent of *x*, is the *β* value given by eq (117) on the boundary and at the temperature in question. $\left({\bar{H}}_{1}^{\beta +\delta }-H_{1}^{0}\right)/T$ was calculated from eq (4) from ${\bar{S}}_{2}^{\beta +\delta }$, $\left({\bar{G}}_{1}^{\beta +\delta }-G_{1}^{0}\right)/T$, and $S_{1}^{0}$ (eq (45)). $C_{p}^{0\left(\beta +\delta \right)}$ is the total derivative of eq (98) with respect to temperature (with *x* but not *x_β_* independent of temperature); by eq (5) this may be broken up into the partial molal properties:

$${\left({\bar{C}}_{p}\right)}_{1}^{\beta +\delta }={\left[\partial \left(H^{0\left(\beta \right)}-H_{298.15}^{0\left(\alpha +\delta \right)}\right)/\partial T\right]}_{x_{\beta }}+{\left[\partial \left(H^{0\left(\beta \right)}-H_{298.15}^{0\left(\alpha +\delta \right)}\right)/\partial x\right]}_{x_{\beta }}\left[dx_{\beta }/dT\right]-\frac{1}{2}x_{\beta }d\left[\left({\bar{H}}_{2}^{\beta +\delta }-H_{2}^{0}\right)+\left(H_{2}^{0}-H_{2\left(298\right)}^{0}\right)\right]/dT-\frac{1}{2}\left[\left({\bar{H}}_{2}^{\beta +\delta }-H_{2}^{0}\right)-{\left({\bar{H}}_{2}^{\alpha +\delta }-H_{2}^{0}\right)}_{298}+\left(H_{2}^{0}-H_{2\left(298\right)}^{0}\right)\right]\left[dx_{\beta }/dT\right];$$ (119)

$${\left({\bar{C}}_{p}\right)}_{2}^{\beta +\delta }=d\left[\left({\bar{H}}_{2}^{\beta +\delta }-H_{2}^{0}\right)+\left(H_{2}^{0}-H_{2\left(298\right)}^{0}\right)\right]/dT.$$ (120)

(The quantities needed in eqs (119) and (120) can be obtained from eqs (28), (29), (92), (99), and (105).)

### 6.5. Other *δ* Properties

The remaining properties in the small part of the *δ* field considered here (from *T*=790 to 850, and from *x*=1.171 to 1.25) were derived as follows. From eqs (90), (103), and (44),

$$\begin{array}{l} x=1.17\;\text{to}\;1.4,\;T=790\;\text{to}\;850:\left({\bar{H}}_{1}^{\delta }-H_{1}^{0}\right)/T\\ \;=9.0646-5.3113\left(10^{-3}\right)T+4.4945\left(10^{-6}\right)T^{2}-1.6785\left(10^{-9}\right)T^{3}-3114.0/T. \end{array}$$ (121)

The following boundary points were selected as giving the best overall continuity of properties at the points:

$$\begin{array}{l} x=1.25:\delta /\left(\alpha +\delta \right)\;\text{boundary},\\ T_{1}=791.5;\delta /\left(\beta +\delta \right)\;\text{boundary},T_{2}=845.6. \end{array}$$ (122)

At these points eqs (69) and (100) give respectively

$$\begin{array}{l} {\left[\left({\bar{G}}_{2}^{\alpha +\delta }-G_{2}^{0}\right)/T\right]}_{791.5,1.25}=-20.0567;\\ \;{\left[\left({\bar{G}}_{2}^{\beta +\delta }-G_{2}^{0}\right)/T\right]}_{845.6,1.25}=-16.3872. \end{array}$$ (123)

However, the integration between these two temperatures of eq (11), after substitution from eq (104) gives a corresponding increment of $\left({\bar{G}}_{2}^{\delta }-G_{2}^{0}\right)/T$ which is 0.0203 Jess than that of eq (123); but by adjusting the integration constant so that the discrepancy has at the two points the same magnitude but opposite signs, the same equation then gives

$${\left[\left({\bar{G}}_{2}^{\delta }-G_{2}^{0}\right)/T\right]}_{820,1.25}=-18.0684.$$ (124)

From eqs (67) and (102)

$${\left[\left({\bar{G}}_{2}^{\delta }-G_{2}^{0}\right)/T\right]}_{820,1.171}=-18.2174.$$ (125)

By making the reasonable assumption that in this small composition range $a_{2}^{\delta }$ is proportional to some constant power of *x*, eqs (124) and (125) give:

$$x=1.17\;\text{to}\;1.25:{\left[\left({\bar{G}}_{2}^{\delta }-G_{2}^{0}\right)/T\right]}_{820}=5.2281\;\log\;x-18.5751.$$ (126)

After substitution from eq (104), the general integration of eq (11) so as to satisfy eq (126) then gives:

$$\begin{array}{l} x=1.17\;\text{to}\;1.25,T=790\;\text{to}\;850:\left({\bar{G}}_{2}^{\delta }-G_{2}^{0}\right)/T\\ \;=5.2281\;\log\;x-15.2606-39546.3/T+15.33532\;\log\;T+2.678\left(10^{-4}\right)T+6000/T^{2}. \end{array}$$ (127)

From eqs (90), (103), and (44),

$$\begin{array}{l} x=1.17\;\text{to}\;1.4,T=790\;\text{to}\;850:\left({\bar{H}}_{1}^{\delta }-H_{1}^{0}\right)/T\\ \;=9.0646-5.3113\left(10^{-3}\right)T+4.4945\left(10^{-6}\right)T^{2}-1.6785\left(10^{-9}\right)T^{3}-3114.0/T. \end{array}$$ (128)

From eqs (102) and (115) we have

$${\left[\left({\bar{G}}_{1}^{\delta }-G_{1}^{0}\right)/T\right]}_{820,1.171}=-0.1292.$$ (129)

Equations (126) and (129) may be substituted into the Gibbs-Duhem equation (eq (9)), the integration from *x*=1.171 to higher *x* giving

$$x=1.17\;\text{to}\;1.25:{\left[\left({\bar{G}}_{1}^{\delta }-G_{1}^{0}\right)/T\right]}_{820}=1.1997-1.13526\;x.$$ (130)

When eq (128) is substituted into eq (11) and the latter is then integrated so as to satisfy eq (130), there is obtained

$$\begin{array}{l} x=1.17\;\text{to}\;1.25,T=790\;\text{to}\;850:\left({\bar{G}}_{1}^{\delta }-G_{1}^{0}\right)/T\\ \;=-1.13526x+62.6621-3114.0/T-20.87215\;\log\;T+5.3113\left(10^{-3}\right)T-2.24725\left(10^{-6}\right)T^{2}+5.595\left(10^{-10}\right)T^{3}. \end{array}$$ (131)

The partial molal entropies can be derived from eq (4). Substitution from eqs (45), (128), and (131) gives ${\bar{S}}_{1}^{\delta }$, and substitution from eqs (95), (104), and (127) gives ${\bar{S}}_{2}^{\delta }$:

$$\begin{array}{l} x=1.17\;\text{to}\;1.25,T=790\;\text{to}\;850:{\bar{S}}_{1}^{\delta }\\ \;=30.19860\;\log\;T+1.13526\;x-70.0120; \end{array}$$ (132)

$$\begin{array}{l} x=1.17\;\text{to}\;1.25,T=790\;\text{to}\;850:{\bar{S}}_{2}^{\delta }\\ \;=1.7718-5.2281\;\log\;x. \end{array}$$ (133)

The substitution of eqs (132) and (133) into eq (5) gives the entropy:

$$\begin{array}{l} x=1.17\;\text{to}\;1.25,T=790\;\text{to}\;850:S{{}^{\circ}}^{\left(\delta \right)}\\ =30.19860\;\log\;T+2.02116\;x-2.61405\;x\;\log\;x-70.0120. \end{array}$$ (134)

^16^

For the composition *x*=1.25 the discrepancy at each adopted phase-field boundary between the final equations for the *δ* and the two-phase fields is given in table 6 for each of the five properties which should have identical values. (In each such case, the average value is tabulated.) Since continuity was required at the boundary at *x*=1.171, presumably the discrepancies would be small at the boundaries for all intervening values of *x.*

### 6.6. Discussion of the *δ* Phase-Field Boundaries

The *δ/(α+δ*) and *δ/(β+δ*) boundaries have been drawn in figure 1 through the adopted *δ* eutectoid point (eq (102)) and the adopted temperatures for *x*=1.25 (eq (122)). Though no effort is made in this paper to derive consistent thermodynamic properties for higher values of *x*, these two boundaries have been extended in figure 1 in a reasonable but empirical way. In particular, the ensemble of experimental points shown for the *δ/(β+δ*) boundary shows wide discrepancies,^17^ but the boundary drawn has been made to pass through the “hydrogen-pressure” values of Edwards et al. [5] and of LaGrange et al. [8]. The points of LaGrange et al. indicate some curvature in the boundary, but those of Edwards et al. (at seven temperatures from 973 to 1,148 °K) show a vertical boundary within the experimental error. In a previous paper [17] the author criticized a vertical boundary on thermodynamic grounds which would lead one to predict that it has a positive finite slope. The same argument still holds, but may be reexamined somewhat more quantitatively.

The hydrogen activity in a one-phase field *b* is of course a function of both temperature and composition, and its variation with temperature along the boundary *x_b_*(*T*) separating the *b* and (*b*+*c*) fields may be written as a total derivative:

$$d\;\ln\;a_{2}/dT={\left(\partial \;\ln\;a_{2}/\partial T\right)}_{x}+{\left(\partial \;\ln\;a_{2}/\partial x\right)}_{T}/\left(dT/dx_{b}\right),$$ (135)

or in the present case

$$d\;\ln\;a_{{\;}_{2}}^{b+c}/dT={\left(\partial \;\ln\;a_{{\;}_{2}}^{b}/\partial T\right)}_{x_{b}}+{\left(\partial \;\ln\;a_{2}^{b}/\partial x\right)}_{T}/(dT/dx_{b}).$$ (136)

Substituting for the first two derivatives from eqs (10) and (11), eq (136) becomes

$${\left(\partial \;\ln\;a_{2}^{b}/\partial x\right)}_{T}={\left(RT^{2}\right)}^{-1}(dT/dx_{b})[({\bar{H}}_{2}^{b}-H_{2}^{{}^{\circ}})-({\bar{H}}_{2}^{b+c}-H_{2}^{{}^{\circ}})].$$ (137)

If the thermodynamic properties as formulated are consistent, regardless of how near to correct they are, they must satisfy eq (137) along every phase-field boundary. For example, the equation may be applied to the *δ/(α+δ*) and *δ/(β+δ*) boundaries at *x*=1.25. In the former case the bracketed quantity in eq (137) is −3006 (from eqs (56) and (104)), and in the latter case it is +4577 (from eqs (99) and (104)). Since, as expected, (∂ ln *α*_2_*^δ^/*∂*x)_T_* is positive in both cases (eq (127)), the slopes of the *δ/(α+δ*) and *δ/(β+δ*) boundaries at this composition will thus be negative and positive respectively, as they are in figure 1.

The measured *δ*-phase isotherms of hydrogen pressure [3,5,8] are in genera] considerably steeper than indicated by eq (127), according to which $a_{2}^{\delta }$ is at constant temperature approximately proportional to x^1.14^ (up to *x*=1.25). It may be noted furthermore that the isotherms cited usually show a more gradual change in slope,^18^ as the hydrogen content changes and the sample crosses a phase-field boundary, when the latter is contiguous with the *δ* field than when it is contiguous with some other one-phase field. If, as seems to be the case, the heats of hydriding do not change rapidly with temperature, eq (137) indicates that at values of *x* considerably greater than 1.25 the far steeper *δ/(β+δ*) boundary indicates a much greater value of ${\left(\partial \;\ln\;a_{2}^{\delta }/\partial x\right)}_{T}$ than at lower values of *x*, as experiment indicates. By the same reasoning, the *δ/(α+δ*) boundary would be expected to become much steeper as *x* increases. This boundary in figure 1 was drawn to pass through the points indicated by thermal-expansion studies from 293 to 673 °K [12,14], and does become somewhat steeper. If this boundary were assumed to be an equilibrium one, it would be possible to draw some conclusions about the *δ*-field hydrogen activities at temperatures too low for direct accurate measurements.

A sample such as ZrH_1.25_ shows the interesting behavior of being in the one-phase *δ* field at some temperatures, but in a two-phase field at higher or lower temperatures. According to equations derived earlier (5, 50, 51, 54, 55, 103, 119, and 120), the equilibrium heat capacity increases discontinuously as the sample passes from the *δ*-field into either contiguous two-phase field, because a new phase of different energy starts to form at a finite rate. A simple proof may be given that the heat capacity is necessarily greater, near the boundary, in the two-phase than in the one-phase field. If ′ and ″ indicate respectively the one- and two-phase properties, at the boundary *G*″*=G*′, *S*″*=S*′, and so also *dG*″*/dT=dG*′*/dT* since *S*=−*dG/dT.* But since *inside* the two-phase field *G*″*<G*′, then the *G*″ (*T*) curve for the sample must be more negatively curved than the partly metastable *G*′ (*T*) curve, or *d*^2^*G*″/*dT*^2^ *<d*^2^*G*′*/dT*^2^. Hence *dS*″*/dT>dS*′*/dT*, and since *dS/dT=C_p_/T*, it follows that $C_{p}^{\prime \prime }>C_{p}^{\prime }$.

## 7. The *β* (Hydrogen-Poor) and *(α+β*) Phase Fields

Unlike the thermodynamic properties in the *(α+δ*) and *(β+δ*) fields derived in sections 5 and 6, those in the *(α+δ*) field were derived from the properties of the two adjacent one-phase fields using phase-field boundaries thermodynamically consistent with these properties. This consistency was imposed by finding two boundaries such that at each temperature ${\bar{G}}_{2}^{\alpha }={\bar{G}}_{2}^{\beta }$ and ${\bar{G}}_{1}^{\alpha }={\bar{G}}_{1}^{\beta }$ (eqs (18) and (22)). The *α*-field properties were formulated in section 5, and the *β*-field properties for *x*≧0.570, in section 6. However, since it is assumed in this paper that *x*≦0.570 in all of the (*α*+*β*) field (eq (101) and fig. 1), the pertinent *β*-field properties are those for the lower range of *x*, and are derived in the present section. This includes *β*-zirconium (*x*=0) from its transformation temperature, 1,136°, up to 1,200 °K.

### 7.1. Discussion of Thermodynamic Consistency

The thermodynamic data in the presently considered ranges of temperature and composition which were given greatest weight are as follows. Figure 1 shows, in addition to the *α*/(*α*+*β*) and *β*/(*α*+*β*) boundaries finally arrived at in this paper, various points on these boundaries indicated by three experimental investigations [6, 8, 10]. The points from the two investigations of hydrogen pressure [6, 8] showed general agreement, but the X-ray measurements [10], which did not involve the *α*/(*α*+*β*) boundary, indicated a much higher curve for the *β*/(*α*+*β*) boundary. The compositions of the eutectoid *β* phase reported by these investigators are equivalent to the following values of *x:* Ells and McQuillan [6], 0.47; LaGrange et al. [8], 0.50; Vaughan and Bridge [10], 0.72. Douglas and Victor’s [16] measurements of the enthalpy of Zr(*β*) and of ZrH_0.324_ (*β*), when reduced to a basis of 298.15 °K by eqs (52) and (53), give the following mean values:

$$\begin{array}{l} {\left(H_{1173.2}^{{}^{\circ}\left(\beta \right)}-H_{298.15}^{{}^{\circ}\left(\alpha +\delta \right)}\right)}_{x=0}=7314;\\ \;{\left(H_{1173.2}^{{}^{\circ}\left(\beta \right)}-H_{298.15}^{{}^{\circ}\left(\alpha +\delta \right)}\right)}_{0.324}=9701; \end{array}$$ (138)

in addition, their mean observed values of ${\left(H{{}^{\circ}}^{\left(\beta \right)}-H_{298.15}^{{}^{\circ}\left(\alpha +\delta \right)}\right)}_{0.324}$ were 8836 at *T*= 1073.2 and 8004 + 2^19^ at *T*= 973.2. They actually measured the enthalpy of Zr(*β*) at five temperatures over the very short temperature range 1,153 to 1,173 °K, the values varying linearly with temperature with a heat capacity averaging 9.0. Skinner’s linear heat capacity- temperature equation, which is based on his enthalpy measurements of Zr(*β*) from the transformation temperature to 1,800 °K [22], gives 6.73 in the same temperature range and extrapolates to 6.46 at 1,000 °K.

Development of *β*-phase properties for *x*≦0.570 began with the assumption of the eutectoid temperature *α*- and *β*-compositions already adopted (eqs (47) and (101)), the adopted transformation temperature of zirconium (eq (41)), and the basic relations in sections 2 and 3. In the first trial an enthalpy equation lor the *β* region was formulated in the following way. It was required to satisfy the values of eq (138), to agree at *x*=0.57 in *H* and (∂*H/*∂*x)_T_* with the equation already derived for *x*≧*0.57* (eq (92)), and to correspond to a heat capacity independent of temperature but equaling Skinner’s 1,000 °K value for *x=0* (6.46) and Douglas and Victor’s mean value for *x*=0.324 (8.48).^20^ In the manner outlined later, points on the *α/(α+β*) and *β*/(*α+β*) boundaries at several temperatures were then found by imposing the requirement that the two partial molal free energies be equal.

The *β/*(*α+β*) boundary so found lay considerably above that indicated by the hydrogen-pressure studies of Ells and McQuillan [6] and LaGrange et al. [8] and considerably below that of Vaughan and Bridge [10] (except at *x*=0, where there is general agreement). While the X-ray technique is less sensitive than the hydrogen-pressure method, it seems reasonable to conclude that unless Vaughan and Bridge made a gross misinterpretation of their data their samples actually did correspond to a boundary in the neighborhood of what they reported and that some factor such as unknown impurities accounts for the large differences from the reported hydrogen-pressure curves.^21^ Nevertheless the latter two curves, although they show some differences at the lower temperatures, are in substantial agreement, and seem much more credible. In the author’s derivation of the two boundaries as outlined above, the single requirement that the hydrogen activities be equal $\left({\bar{G}}_{2}^{\alpha }={\bar{G}}_{2}^{\beta }\right)$ gives of course an infinite set of curves, one of which approximated the experimentally observed “hydrogen pressure” boundaries, but at intermediate temperatures with zirconium activities of approximately 0.94 on the *α* boundary and 0.97 on the *β* boundary. Just how sensitive these zirconium activities are to shifts under the experimental conditions of the hydrogen-pressure measurements is not apparent.

It is appropriate of course to examine critically the assumptions and basic data used by the author in deriving the boundaries. The *α*-field activities of hydrogen and zirconium given by eqs (70), (71), and (81), which are related by the exact Gibbs-Duhem equation,^22^ are not only reasonable but are supported by the agreement in table 4 below 820 °K. An independent entropy calculation showing excellent agreement will be made later in this section, and supports the basic hydrogen-activity isotherm assumed (eq (23)). The boundaries are insensitive at the lower temperatures to the heat capacity assumed for *β*-zirconium. The enthalpy measurements on ZrH_0.324_ were of high precision (±5 cal mole^−1^), the sample was of fairly high purity, and there are good reasons for believing that in the temperature range cited above (973 to 1,173 °K) the sample was entirely in the one-pliase *β* field and hence not subject to the uncertainties caused by phase reproportionation. Nor did it seem justified to modify eq (92) for the “hydrogen-rich” *β* enthalpies. In fact, it seems clear that enthalpies corrected additively for small amounts of impurities may still be seriously in error in multiphase fields owing to improper phase compositions, but reliable in one-phase fields. In several attempts it was found impossible to reformulate the *β*-phase enthalpies for *x*≦0.570 with the restrictions mentioned above and still obtain a credible approximately monotonic function which had an appreciable effect on the derived *α*/(*α+β*) and *β*/(*α+β*) boundaries. Furthermore, the choice of a somewhat lower value of the eutectoid *β* composition would do some violence to the fit which led to eq (101) without leading to a much better fit to the experimental “hydrogen-pressure” *β*/(*α+β*) boundaries. This is because the latter suggest a boundary slope near the eutectoid temperature which is several times less steep than that required by eq (137).

### 7.2. The *β* Properties (*x*≦0.57)

In order to derive a thermodynamically consistent set of properties in the range of temperature and composition under consideration (*T*>820, *x*<0.570), it was decided to give approximately equal weight to the hydrogen-pressure results [6, 8] and the enthalpy data [16] by making such changes in the latter as would lead to a derived *β*/(*α+β*) boundary approximately halfway between the experimental “hydrogen-pressure” boundary and that derived above. A new *β*-field enthalpy function was derived by the procedure described earlier except that the heat capacity of Zr(*β*) was raised from 6.46 to 7.76 and that of ZrH_0.324_ (*β*) was raised from 8.48 to 9.00:^23^

$$x≦0.570:\left(H{{}^{\circ}}^{\left(\beta \right)}-H_{298.15}^{{}^{\circ}\left(\alpha +\delta \right)}\right)/T=7.763+2.144x+90.047x^{4}-126.382x^{5}+\left(4990.1x-109834.5x^{4}+147541.6x^{5}-1793.8\right)/T.$$ (139)

From the temperature derivative of eq (139), eqs (6) and (8) give the partial molal heat capacities:

$$x≦0.570:{\left({\bar{C}}_{p}\right)}_{1}^{\beta }=7.763-270.141x^{4}+505.528x^{5};$$ (140)

$$x≦0.570:{\left({\bar{C}}_{p}\right)}_{2}^{\beta }=4.288-270.376x^{3}+1263.82x^{4}.$$ (141)

From eqs (44), (90), and (139):

$$\begin{array}{l} x≦0.570:\left({\bar{H}}_{1}^{\beta }-H_{1}^{{}^{\circ}}\right)/T\\ \;=\left(-215.3+329503.5x^{4}-590166.4x^{5}\right)/T\\ \;+3.7126-270.141x^{4}+505.528x^{5}-5.3113\left(10^{-3}\right)T\\ \;+4.4945\left(10^{-6}\right)T^{2}-1.6785\left(10^{-9}\right)T^{3}. \end{array}$$ (142)

From eqs (26), (28), (29), and (139):

$$\begin{array}{l} x≦0.570:\left({\bar{H}}_{2}^{\beta }-H_{2}^{{}^{\circ}}\right)/T=\left(-2.372+720.376x^{3}-1263.82x^{4}\right)\\ \;+\left(-29528.1-878676x^{3}+1475416x^{4}\right)T\\ \;-2.678\left(10^{-4}\right)T+12000/T^{2}. \end{array}$$ (143)

By substituting from eq (143) in eq (11), integrating to satisfy eq (24), and adding a small correction term “Δ” (a function of *x* only) which will be presently evaluated (eq (151)), there is obtained

$$\begin{array}{l} x≦0.570:\left({\bar{G}}_{2}^{\beta }-G_{2}^{{}^{\circ}}\right)/T\\ \;=6.22313\;\log\;x+4.5758\;\log\;\left(1+1.8493x^{6.97}\right)\\ \;+5.1053+5845.8474x^{3}-10194.2788x^{4}\\ \;+\left(-29528.1-878676x^{3}+1475416x^{4}\right)/T\\ \;+\left(5.46177-1658.73778x^{3}+2910.07193x^{4}\right)\;\log\;T\\ \;+2.678\left(10^{-4}\right)T+6000/T^{2}+\Delta . \end{array}$$ (144)

On substitution from eqs (95), (143), and (144), eq (4) gives (with log (1 + 1.8493*x*^6.97^) expanded to one term):

$$\begin{array}{l} x≦0.570:{\bar{S}}_{2}^{\beta }=-6.22313\;\log\;x-3.675x^{6.97}\\ \;-14.3061-5125.4714x^{3}+8930.4588x^{4}\\ +\left(9.87355+1658.73778x^{3}-2910.07193x^{4}\right)\;\log\;T-\Delta . \end{array}$$ (145)

The entropy will next be found. From eq (45) the entropy of *α*-zirconium at the transformation temperature is

$$S_{1136,x=0}^{0\left(\alpha \right)}=18.7293.$$ (146)

Equations (44) and (139) give for the heat of transformation of zirconium

$${\left(H{{}^{\circ}}^{\left(\beta \right)}-H{{}^{\circ}}^{\left(\alpha \right)}\right)}_{1136,x=0}=937.1.$$ (147)

Adding the corresponding entropy of transformation to eq (146),

$$S_{1136,x=0}^{0\left(\beta \right)}=19.5542.$$ (148)

The entropy at 1,136 °K and any value of *x* up to 0.570, found from eq (7) by integrating from *x*=0 to *x* after substituting from eqs (145) and (148), is

$$\begin{array}{l} x≦0.570:S_{1136}^{0\left(\beta \right)}=19.5542+9.28232x-3.11156x\;\log\;x\\ \;-0.23x^{7.97}-7.1746x^{4}+3.9083x^{5}-\frac{1}{2}\int_{0}^{x}\Delta dx. \end{array}$$ (149)

When, using the heat capacity from eq (139), eq (2) is integrated to satisfy eq (149), the result is

$$\begin{array}{l} x≦0.570:S{{}^{\circ}}^{\left(\beta \right)}=\left(17.87508+4.93677x+207.3422x^{4}-291.0072x^{5}\right)\;\log\;T-35.0610\\ \;-5.80139x-3.11156x\;\log\;x-0.23x^{7.97}\\ \;-640.6838x^{4}+893.0459x^{5}-\frac{1}{2}\int_{0}^{x}\Delta dx. \end{array}$$ (150)

The way in which eq (150) was derived indicates no value of Δ other than zero. With Δ=0, the equation gives for *x*= 0.570 values of entropy which are higher than those of eq (112) by 0.016 at all temperatures. To make the two equations agree, Δ was arbitrarily chosen to be

Δ=−0.13log(x/0.570) (151)

in eqs (144), (145), (149), and (150). Agreement with the value of eq (148) is maintained, and for *x=0.57* eqs (94) and (144) still agree. Since eq (151) changes the calculated values of hydrogen activity by only 3 percent lor *x*=0.2 and by less for higher values of *x*, the agreement with the observed values as tabulated in table 1 is still good and within the experimental error.

Equations (112) and (150) (with Δ=0) both depend on the entropy of zirconium metal at 820 °K, but were otherwise derived by different paths of composition- and temperature-change from this common point. The small disagreement of 0.016 noted above is thus a good check on some of the data entering the derivations, particularly on the basic hydrogen-activity equation (eq (23)) since this equation enters into the derivation of eq (150) only. However, some of the thermal data assumed affect both equations to comparable extents. Specifically, the close agreement is no real check on the *β* enthalpy functions (eqs (139) and (143)), nor on the exact choice of the eutectoid *β* composition (eq (101)), for to these it is rather insensitive.

When eq (144) (with Δ from eq (151)) is substituted into the Gibbs-Duhem relation (eq (9)) and the integration is required to satisfy eq (116), there is obtained

$$\begin{array}{l} x≦0.570:\left({\bar{G}}_{1}^{\beta }-G_{1}^{{}^{\circ}}\right)/T=22.3595-1.32311x\\ \;-2192.19277x^{4}+4077.7115x^{5}-1.607x^{7.97}+1.6x^{14.94}\\ \;+\left(-215.3+329503.5x^{4}-590166.4x^{5}\right)/T\\ \;+\left(-8.54863+622.02667x^{4}-1164.02887x^{5}\right)\;\log\;T\\ \;+5.3113\left(10^{-3}\right)T-2.24725\left(10^{-6}\right)T^{2}\\ \;+5.595\left(10^{-10}\right)T^{3}. \end{array}$$ (152)

For *x*=0 and *T*=1136, this equation gives +0.004. This value should be zero, since 1,136 °K has been assumed to be the transformation temperature of zirconium. ${\bar{S}}_{1}^{\beta }$ for *x*≦0.570 can readily be found from eq (4) or (5) using equations already given (45, 142, and 152).

### 7.3. Derived (*α*+*β*) Boundaries

Points *x_α_* and *x_β_* on the *α*/(*α*+*β*) and *β/*(*α+β*) boundaries as determined by the formulations for the *α* and *β* fields given in this paper were then calculated at several temperatures, and are listed in table 7. This was done by requiring the respective partial molal free energies to be equal at the boundary compositions at the temperature in question (eqs (71), (81), (144), (151), and (152)). The compositions at the eutectoid temperature had already been determined (eqs (47) and (101)). The boundaries meet at the transformation temperature of zirconium. The solid *α*/(*α*+*β*) and *β*/(*α +β*) boundaries in figure 1 were drawn through the points in table 7, except that they were allowed to deviate slightly from the 1,000 °K points, meeting as straight lines at *T*=1136 and *x*=0. LaGrange et ah [8] have pointed out that the two boundaries are required thermodynamically to meet at this point with a difference in slope equal to the heat of transformation of zirconium divided by *RT*^2^. In the notation of the present paper this relation is

$${\left(\frac{dx_{\alpha }}{dT}-\frac{dx_{\beta }}{dT}\right)}_{1136,x=0}={\left(\frac{H^{0\left(\beta \right)}-H^{0\left(\alpha \right)}}{RT^{2}}\right)}_{1136,x=0},$$ (153)

and is true provided the activity of diatomic hydrogen is proportional to *x*^2^ in both solid solutions. Despite eq (23), this is a reasonable assumption for very dilute solutions, and the two boundaries have been drawn in figure 1 so as to meet with slopes satisfying eq (153) with the heat of transformation of eq (147). At the higher temperatures the procedure of this section in defining the boundaries becomes increasingly sensitive to errors in the data, and no calculations of this nature were attempted above 1,000 °K.

### 7.4. The (*α*+*β*) Properties

The values of $\left({\bar{G}}_{1}^{\alpha +\beta }-G_{1}^{0}\right)/T$ and $\left({\bar{G}}_{2}^{\alpha +\beta }-G_{2}^{0}\right)/T$ are of course the values at the boundaries given by the equations just cited. The enthalpy function and the entropy, $\left(H^{0\left(\alpha +\beta \right)}-H_{298.15}^{0\left(\alpha +\delta \right)}\right)/T$ and *S*^0(^*^α^*^+^*^β^*^)^, are given by eq (16) after substituting from eqs (63), (75), (139), (150), and (151) and table 7. ${\bar{S}}_{2}^{\alpha +\beta }$ and ${\bar{S}}_{1}^{\alpha +\beta }$ were calculated from eqs (6) and (5) respectively. $\left({\bar{H}}_{1}^{\alpha +\beta }-H_{1}^{0}\right)/T$ and $\left({\bar{H}}_{2}^{\alpha +\beta }-H_{2}^{0}\right)/T$ were then obtained from eq (4). The latter function was also calculated from eq (26), which takes the form

$$\begin{array}{l} \left({\bar{H}}_{2}^{\alpha +\beta }-H_{2}^{0}\right)/T=\left[2/T\left(x_{\beta }-x_{\alpha }\right)\right]\left[{\left(H^{0\left(\alpha \right)}-H_{298}^{0\left(\alpha +\delta \right)}\right)}_{x_{\beta }}-{\left(H^{0\left(\alpha \right)}-H_{298}^{0\left(\alpha +\delta \right)}\right)}_{x_{\beta }}\right]\\ +{\left({\bar{H}}_{2}^{\alpha +\delta }-H_{2}^{0}\right)}_{298}/T-\left(H_{2}^{0}-H_{2\left(298\right)}^{0}\right)/T. \end{array}$$ (154)

The values calculated by the two methods differed (owing to small computational inconsistencies) by 0.007 on the average, and the mean was tabulated.^24^

The heat capacity in the (*α*+*β*) field was formulated by substituting the *α* and *β* enthalpies (given by eqs (63) and (139) into eq (16) and then taking the total derivative with respect to temperature. For simplicity replacing ${\left(H^{0\left(\alpha \right)}-H_{298}^{0\left(\alpha +\delta \right)}\right)}_{x_{\alpha }}$ by *“H^α^”* and ${\left(H^{0\left(\beta \right)}-H_{298}^{0\left(\alpha +\delta \right)}\right)}_{x_{\beta }}$ by *“H^β^”* and omitting the subscripts *x_α_* and *x_β_* the result is

$$\begin{array}{l} C_{p}^{0\left(\alpha +\beta \right)}={\left(\frac{\partial H^{\alpha +\beta }}{\partial T}\right)}_{x}\\ \;=\left[\frac{x_{\beta }-x}{x_{\beta }-x_{\alpha }}\right]\left[\frac{\partial H_{\alpha }}{\partial T}+\left(\frac{\partial H_{\alpha }}{\partial x}-\frac{H^{\beta }-H^{\alpha }}{x_{\beta }-x_{\alpha }}\right)\frac{dx_{\alpha }}{dT}\right]\\ \;+\left[\frac{x-x_{\alpha }}{x_{\beta }-x_{\alpha }}\right]\left[\frac{\partial H_{\beta }}{\partial T}+\left(\frac{\partial H^{\beta }}{\partial x}-\frac{H^{\beta }-H^{\alpha }}{x_{\beta }-x_{\alpha }}\right)\frac{dx_{\beta }}{dT}\right]. \end{array}$$ (155)

The phase-field boundary slopes in eq (155) may be replaced in terms of more common thermodynamic functions. Since

$${\bar{G}}_{2}^{\alpha +\beta }={\bar{G}}_{2}^{\alpha }={\bar{G}}_{2}^{\beta },$$ (156)

we have

$$\frac{d\left({\bar{G}}_{2}^{\alpha +\beta }-G_{2}^{0}\right)/T}{dT}=\frac{\partial \left({\bar{G}}_{2}^{b}-G_{2}^{0}\right)/T}{dT}+\frac{\partial \left({\bar{G}}_{2}^{b}-G_{2}^{0}\right)/T}{dT}\frac{dx_{b}}{dT},$$ (157)

where *b* is either *α* or *β*. *dx_α_/dT* and *dx_β_/dT* may be substituted into eq (155) from eqs (157). If the temperature derivatives appearing in eq (157) are replaced from eq (11), some simplification is achieved by substituting for the resulting partial molal enthalpies from eqs (26) and (154). Equation (155) fin all v becomes

$$\begin{array}{l} C_{p}^{0\left(\alpha +\beta \right)}=\left[\frac{x_{\beta }-x}{x_{\beta }-x_{\alpha }}\right]\left[\frac{\partial H^{\alpha }}{\partial T}+2{\left(\frac{\partial H^{\alpha }}{\partial x}-\frac{H^{\beta }-H^{\alpha }}{x_{\beta }-x_{\alpha }}\right)}^{2}/T^{2}\frac{\partial }{\partial x}\left(\frac{\bar{G_{2}^{\alpha }-G_{2}^{0}}}{T}\right)\right]\\ +\left[\frac{x-x_{\alpha }}{x_{\beta }-x_{\alpha }}\right]\left[\frac{\partial H^{\beta }}{\partial T}+2{\left(\frac{\partial H^{\beta }}{\partial x}-\frac{H^{\beta }-H^{\alpha }}{x_{\beta }-x_{\alpha }}\right)}^{2}/T^{2}\frac{\partial }{\partial x}\left(\frac{{\bar{G}}_{2}^{\beta }-G_{2}^{0}}{T}\right)\right]. \end{array}$$ (158)

The partial molal heat capacities can be found from eq (158) by using eq (5).

The procedure outlined in this section is generally applicable to determining the phase-field boundaries and the thermodynamic properties of two-phase fields from the properties of the adjoining one-phase fields. In the present case, there is considerable uncertainty in the (*α*+*β*) properties because of uncertainty in the exact locations and shapes of the boundaries, and this is particularly true of the heat capacity, which eq (155) shows to be highly dependent on the slopes of the boundaries. However, as noted earlier, small changes in the one-phase properties often have large effects on the calculated boundaries. For this reason, due consideration of direct evidence as to the positions of the boundaries can serve in a treatment of the present type to define the properties of the neighboring one-phase fields more accurately.

## 8. Tables of Thermodynamic Functions

The common integral and differential thermodynamic properties were calculated as described previously in this paper, and are listed in tables 8–14 for zirconium and several compositions of zirconium hydride (ZrH*_x_*). In addition, table 15 gives the properties of ideal “normal” hydrogen gas (H_2_, 25% para and 75% ortho) [20], which are closely related to those of the Zr-H system. The tables cover evenly spaced compositions of ZrH*_x_* with *x*=0, 0.25, 0.50, 0.75, 1.00, and 1.25; in addition, the composition *x*=0.57 is also included in the tabulation, partly because this composition is of special interest as the assumed *β*-phase eutectoid composition, and partly because the *β*-field properties are separately formulated on both sides of this composition. In conformity with the conventions used throughout this paper, temperatures are in degrees Kelvin and the thermodynamic properties are given in defined thermochemical calories per degree per mole of ZrH*_x_* (or H_2_, in table 15). The temperatures tabulated, which begin with 298.15 °K, run every 50° from 300 to 1,200 °K except that the eutectoid temperature, 820 °K, and the transformation temperature, 1,136 °K, are included in all the tables because of some interest in these particular isotherms. In addition, each table includes the temperature at which the derived properties indicate that the particular composition crosses a phase-field boundary, with properties being tabulated for both the fields which meet at that boundary. Except for the properties of hydrogen gas and for those of *α*-zirconium which were not derived from those of the zirconium hydrides, the values of the properties have been rounded off to two decimal places as being more in keeping with their uncertainties in general.

## Figures and Tables

**Table 1:** Deviation from eg (23) (or (24)) of observed hydrogen activities of β-*ZrH_x_* at 1,073.2 °K

Reference	*x*	$\frac{a_{2}\left(\text{obs}\right)-a_{2}\left(\text{calcd}\right)}{a_{2}\left(\text{obs}\right)}\times 100$

[8]	0.05	+43
[8]	.11	−12
[8]	.19	0
[8]	.27	0
[8]	.37	−1
[8]	.46	0
[8]	.55	+1
[8]	.64	0
[5]	.650	−4
[5]	.733	0
[8]	.76	0
[5]	.815	+4
[8]	.87	0
[5]	.894	+5
[8]	.92	−4
[8]	.96	0
[8]	1.00	−8

**Table 2:** Deviation of eq (44) from observed values of enthalpy of α-zirconium

Temperature	$H_{1}^{{}^{\circ}}-H_{1\left(273.2\right)}^{{}^{\circ}}$ Mean observed	Mean observed—calculated enthalpy

*°K*	*cal mole*^−1^	*cal mole*^−1^
273.2	(0)	(0)
373. 2	630	+ 1
473.2	1284	− 9
573.2	1989	+ 8
673.2	2686	− 1
773.2	3409	0
873.2	4147	− 2
973.2	4923	+ 9
1073.2	5708	− 7
1123.2	6133	+ 1

**Table 3:** Isotherms of enthalpy in the (α+δ) field of Zr–H (In cal mole^−1^ of ZrH_x_)

*T*	*A*′ in eq (49)	*B*′ in eq (49)

*°K*		
273. 15	0	0
373. 15	637. 5	114. 0
473. 15	1305. 6	356. 5
573. 15	2041. 8	694. 9
673. 15	2849. 4	1039. 7
773. 15	3794. 2	1410. 0
823. 15	4277. 0	1705. 8

**Table 4:** The compositions of the *α*/(*α*+δ) boundary by two methods

*T*	*x*_α_ at boundary	
	Equality of hydrogen activity	Equality of enthalpy

°*K*		
298.15	0.000015	0.0006
300	.000016	.0006
350	.00010	.0010
400	.00043	.0010
450	.0013	.0013
500	.0031	.0025
550	.0064	.0053
600	.0114	.0102
650	.0188	.0178
700	.0288	.0282
750	.0418	.0414
800	.0578	.0576
820	.0650	.0650

**Table 5:** Deviation from eq (92) of the mean observed enthalpies (For each temperature and composition the first value is the mean observed $\left(H^{0\left(\beta \right)}-H_{298.15}^{0\left(\alpha +\delta \right)}\right)$ and the value in parentheses is the observed — calculated, both in cal/mole of ZrH*_x_*.)^13^

*T*	*x*=0.556	*x*=0.701	*x*=0.999	*x*=1.071

°K				
923.2	8291 ±6(−44)	8876 ±0(+36)		
973.2	8839 (−39)	9423 (+24)		
1073.2	9971 (+8)	10528 (+13)		[11906(−16)]
1123.2			[12211(−27)]	12520(0)
1173.2	11040 (−9)	11640 (+8)	12828(0)	13118(0)

**Table 6:** Discrepancies of formulated properties of *ZrH_1.25_* at the adopted phase-field boundaries

Property	″*δ*″−″ (*α+δ*) ″ at 791.5 °K	″*δ*″−″(*β+δ*)″ at 845.6 °K

$\left(H{}^{\circ}-H_{298.15}^{{}^{\circ}\left(\alpha +\delta \right)}\right)/T$	+0.005	+0.007
S°	+ .002	+ .008
$-\left(G{}^{\circ}-H_{298.15}^{{}^{\circ}\left(\alpha +\delta \right)}\right)/T$	− .002	+ .001
$\left({\bar{G}}_{1}-G_{1}^{{}^{\circ}}\right)/T$	− .006	+ .006
$\left({\bar{G}}_{2}-G_{2}^{{}^{\circ}}\right)/T$	+ .010	− .010

**Table 7:** Calculated boundary compositions of the (*α*+*β*) phase field

*T*	*x_α_*	*x_β_*

*°K*		
820	0.0650	0.570
850	.0696	.535
865.3	.0717	.500
873.2	.0727	.472
900	.0747	.355
950	.0745	.259
956.6	.0741	.250
1000	.0708	.202
1136	.0000	.000

**Table 8:** Thermodynamic functions for zirconium, *Zr* T in deg K, thermodynamic functions in cal (deg K)^−1^ mole^−1^. Subscript 1 refers to Zr(*α*). Subscript 2 refers to H_2_(g). ${\left({\bar{C}}_{p}\right)}_{1}=C_{p^{{}^{\circ}}}\;{\bar{S}}_{1}=S^{{}^{\circ}}\;{\bar{S}}_{2}=-\left({\bar{G}}_{2}-G_{2}^{{}^{\circ}}\right)/T=\infty$

*T*	Phases present	$\frac{H^{\circ }-H_{298.15}^{\circ }}{T}$	$C_{p}^{\circ }$	*S*°	$\frac{G^{{}^{\circ}}-H_{298.15}^{{}^{\circ}}}{T}$	$\frac{{\bar{H}}_{1}^{{}^{\circ}}-H{{}^{\circ}}_{1}}{T}$	$\frac{{\bar{G}}_{1}-G_{1}^{{}^{\circ}}}{T}$	$\frac{{\bar{H}}_{2}-H_{2}^{{}^{\circ}}}{T}$	${\left({\bar{C}}_{p}\right)}_{2}$

298.15	*α*	0	6.197	9.290	−9.290	0	0	−86.12	1.16
300	*α*	0.038	6.205	9.329	−9.291	0	0	−85.62	1.20
350	*α*	.934	6.404	10.301	−9.367	0	0	−74.13	2.18
400	*α*	1.628	6.572	11.167	−9.539	0	0	−65.39	3.10
450	*α*	2.186	6.712	11.949	−9.763	0	0	−58.49	3.97
500	*α*	2.644	6.830	12.663	−10.019	0	0	−52.90	4.78
550	*α*	3.030	6.931	13.319	−10.289	0	0	−48.25	5.52
600	*α*	3.359	7.020	13.926	−10.567	0	0	−44.32	6.21
650	*α*	3.643	7.102	14.491	−10.848	0	0	−40.95	6.84
700	*α*	3.893	7.182	15.020	−11.127	0	0	−38.02	7.40
750	*α*	4.115	7.265	15.519	−11.404	0	0	−35.44	7.91
791.5	*α*	4.282	7.340	15.912	−11.629	0	0	−33.53	8.29
800	*α*	4.315	7.357	15.990	−11.675	0	0	−33.16	8.36
820	*α*	4.390	7.396	16.172	−11.782	0	0	−32.32	8.53
845.6	*α*	4.482	7.451	16.400	−11.919	0	0	−31.30	8.64
850	*α*	4.497	7.461	16.439	−11.942	0	0	−31.13	8.66
865.3	*α*	4.550	7.496	16.573	−12.023	0	0	−30.55	8.73
891.5	*α*	4.637	7.561	16.797	−12.100	0	0	−29.60	8.85
900	*α*	4.665	7.584	16.869	−12.204	0	0	−29.31	8.89
950	*α*	4.822	7.729	17.283	−12.461	0	0	−27.67	9.11
956.6	*α*	4.842	7.751	17.337	−12.494	0	0	−27.46	9.14
1000	*α*	4.972	7.904	17.684	−12.712	0	0	−26.18	9.34
1050	*α*	5.116	8.111	18.074	−12.958	0	0	−24.83	9.56
1080.9	*α*	5.204	8.258	18.312	−13.108	0	0	−24.05	9.70
1100	*α*	5.258	8.357	18.457	−13.199	0	0	−23.59	9.79
1136	*α*	5.359	8.560	18.729	−13.370	0	0	−22.76	9.95
1136	*β*	6.18	7.76	19.55	−13.37	0.82	0	−28.66	4.29
1150	*β*	6.20	7.76	19.65	−13.45	.80	−0.01	−28.35	4.29
1200	*β*	6.27	7.76	19.98	−13.71	.73	−.04	−27.29	4.29

**Table 9:** Thermodynamic functions for *ZrH_0.25_* *T* in deg K, thermodynamic functions in cal (deg K)^−1^ mole^−1^. Subscript 1 refers to Zr(*α*). Subscript 2 refers to H_2_(g).

*T*	Phases present	$\frac{H^{\circ }-H_{298.15}^{\circ }}{T}$	$C_{p}^{\circ }$	*S*°	$\frac{G^{{}^{\circ}}-H_{298.15}^{{}^{\circ}}}{T}$	$\frac{{\bar{H}}_{1}-H_{1}^{{}^{\circ}}}{T}$	${\left({\bar{C}}_{p}\right)}_{1}$	${\bar{S}}_{1}$	$\frac{{\bar{G}}_{1}-G_{1}^{{}^{\circ}}}{T}$	$\frac{{\bar{H}}_{2}-H_{2}^{{}^{\circ}}}{T}$	${\left({\bar{C}}_{p}\right)}_{2}$	${\bar{S}}_{2}$	$\frac{{\bar{G}}_{2}-G_{2}^{{}^{\circ}}}{T}$

298.15	*α+δ*	0	6.54	9.27	−9.27	0.02	6.32	9.31	−0.00	−139.12	1.71	−0.29	−107.62
300	*α+δ*	0.04	6.54	9.31	−9.27	.02	6.32	9.34	−.00	−138.29	1.74	−.28	−106.76
350	*α+δ*	.98	6.78	10.33	−9.35	.02	6.42	10.33	−.00	−119.20	2.86	+.07	−86.95
400	*α+δ*	1.73	7.11	11.26	−9.53	.02	6.58	11.19	−.00	−104.73	4.24	.54	−72.02
450	*α+δ*	2.35	7.51	12.12	−9.77	.03	6.81	11.98	−.00	−93.32	5.57	1.12	−60.36
500	*α+δ*	2.89	7.94	12.93	−10.05	.04	7.12	12.71	−.00	−84.07	6.56	1.76	−51.03
550	*α+δ*	3.37	8.39	13.71	−10.35	.08	7.51	13.41	−.01	−76.44	7.05	2.41	−43.38
600	*α+δ*	3.80	8.85	14.46	−10.66	.13	7.97	14.08	−.02	−70.06	7.03	3.03	−37.01
650	*α+δ*	4.21	9.32	15.19	−10.98	.21	8.48	14.74	−.04	−64.69	6.75	3.58	−31.62
700	*α+δ*	4.59	9.86	15.90	−11.30	.31	9.01	15.39	−.06	−60.09	6.76	4.08	−27.00
750	*α+δ*	4.97	10.53	16.60	−11.63	.43	9.53	16.03	−.08	−56.07	8.00	4.58	−23.00
800	*α+δ*	5.34	11.48	17.31	−11.97	.55	10.00	16.66	−.11	−52.41	11.86	5.20	−19.49
820	*α+δ*	5.50	11.99	17.60	−12.10	.61	10.17	16.91	−.13	−50.98	14.54	5.52	−18.22
820	*α+β*	6.49	9.99	18.59	−12.10	.26	7.56	16.56	−.13	−40.26	19.43	16.24	−18.22
850	*α+β*	6.64	11.60	18.97	−12.34	.24	6.88	16.82	−.14	−38.15	37.74	17.19	−16.81
900	*α+β*	7.18	17.32	19.90	−12.73	+.07	3.96	17.09	−.15	−31.22	106.89	22.53	−14.80
950	*α+β*	7.61	14.82	20.74	−13.13	−.08	5.26	17.35	−.15	−25.53	76.49	27.08	−13.27
956.6	*α+β*	7.66	14.94	20.84	−13.18	−.10	5.29	17.39	−.15	−24.86	77.14	27.62	−13.10
956.6	*β*	7.66	8.53	20.84	−13.18	+1.23	7.20	18.72	−.15	−35.49	10.61	16.99	−13.10
1000	*β*	7.70	8.53	21.22	−13.52	1.15	7.20	19.04	−.20	−33.80	10.61	17.46	−11.56
1050	*β*	7.74	8.53	21.64	−13.90	1.06	7.20	19.39	−.25	−32.03	10.61	17.98	−9.96
1100	*β*	7.77	8.53	22.03	−14.26	0.96	7.20	19.72	−.30	−30.42	10.61	18.47	−8.50
1136	*β*	7.80	8.53	22.31	−14.51	.89	7.20	19.95	−.33	−29.35	10.61	18.82	−7.54
1150	*β*	7.80	8.53	22.41	−14.61	.86	7.20	20.04	−.34	−28.96	10.61	18.95	−7.18
1200	*β*	7.84	8.53	22.77	−14.94	.76	7.20	20.35	−.38	−27.61	10.61	19.40	−5.98

**Table 10:** Thermodynamic functions for *ZrH_0.50_* *T* in deg K, thermodynamic functions in cal (deg K)^−1^ mole^−1^. Subscript 1 refers to Zr(*α*). Subscript 2 refers to H_2_(g).

*T*	Phases present	$\frac{H^{\circ }-H_{298.15}^{\circ }}{T}$	$C_{p}^{\circ }$	*S*°	$\frac{G^{{}^{\circ}}-H_{298.15}^{{}^{\circ}}}{T}$	$\frac{{\bar{H}}_{1}-H_{1}^{{}^{\circ}}}{T}$	${\left({\bar{C}}_{p}\right)}_{1}$	${\bar{S}}_{1}$	$\frac{{\bar{G}}_{1}-G_{1}^{{}^{\circ}}}{T}$	$\frac{{\bar{H}}_{2}-H_{2}^{{}^{\circ}}}{T}$	${\left({\bar{C}}_{p}\right)}_{2}$	${\bar{S}}_{2}$	$\frac{{\bar{G}}_{2}-G_{2}^{{}^{\circ}}}{T}$

298.15	*α+δ*	0	6.75	9.23	−9.23	0.02	6.32	9.31	−0.00	−139.12	1.71	−0.29	−107.62
300	*α+δ*	0.04	6.76	9.28	−9.23	.02	6.32	9.34	−.00	−138.29	1.74	−.28	−106.76
350	*α+δ*	1.03	7.14	10.34	−9.32	.02	6.42	10.33	−.00	−119.20	2.86	+.07	−86.95
400	*α+δ*	1.82	7.64	11.33	−9.51	.02	6.58	11.19	−.00	−104.73	4.24	.54	−72.02
450	*α+δ*	2.50	8.21	12.26	−9.76	.03	6.81	11.98	−.00	−93.32	5.57	1.12	−60.36
500	*α+δ*	3.10	8.76	13.15	−10.06	.04	7.12	12.71	−.00	−84.07	6.56	1.76	−51.03
550	*α+δ*	3.64	9.27	14.01	−10.38	.08	7.51	13.41	−.01	−76.44	7.05	2.41	−43.38
600	*α+δ*	4.12	9.73	14.84	−10.72	.13	7.97	14.08	−.02	−70.06	7.03	3.03	−37.01
650	*α+δ*	4.57	10.17	15.64	−11.06	.21	8.48	14.74	−.04	−64.69	6.75	3.58	−31.62
700	*α+δ*	4.99	10.70	16.41	−11.42	.31	9.01	15.39	−.06	−60.09	6.76	4.08	−27.00
750	*α+δ*	5.40	11.53	17.17	−11.78	.43	9.53	16.03	−.08	−56.07	8.00	4.58	−23.00
800	*α+δ*	5.82	12.97	17.96	−12.14	.55	10.00	16.66	−.11	−52.41	11.86	5.20	−19.49
820	*α+δ*	6.00	13.80	18.29	−12.28	.61	10.17	16.91	−.13	−50.98	14.54	5.52	−18.22
820	*α+β*	8.34	12.42	20.62	−12.28	.26	7.56	16.56	−.13	−40.26	19.43	16.24	−18.22
850	*α+β*	8.54	16.32	21.12	−12.59	.24	6.88	16.82	−.14	−38.15	37.74	17.19	−16.81
865.3	*α+β*	8.72	22.57	21.46	−12.74	.22	5.74	16.94	−.14	−36.72	67.33	18.09	−16.14
865.3	*β*	8.72	10.51	21.46	−12.74	2.55	6.68	19.26	−.14	−46.02	15.35	8.78	−16.14
900	*β*	8.79	10.51	21.87	−13.08	2.41	6.68	19.52	−.24	−43.93	15.35	9.39	−14.37
950	*β*	8.88	10.51	22.44	−13.56	2.24	6.68	19.88	−.37	−4L 19	15.35	10.22	−12.07
1000	*β*	8.96	10.51	22.98	−14.02	2.07	6.68	20.23	−.48	−38.72	15.35	11.00	−10.02
1050	*β*	9.04	10.51	23.49	−14.46	1.91	6.68	20.55	−.57	−36.49	15.35	11.75	−8.19
1100	*β*	9.10	10.51	23.98	−14.88	1.75	6.68	20.86	−.66	−34.46	15.35	12.47	−6.54
1136	*β*	9.15	10.51	24.32	−15.17	1.64	6.68	21.08	−.71	−33.11	15.35	12.97	−5.45
1150	*β*	9.16	10.51	24.45	−15.29	1.59	6.68	21.16	−.73	−32.61	15.35	13.16	−5.05
1200	*β*	9.22	10.51	24.90	−15.68	1.44	6.68	21.44	−.80	−30.92	15.35	13.81	−3.70

**Table 11:** Thermodynamic functions for *ZrH_0.57_* *T* in deg K, thermodynamic functions in cal (deg K)^−1^ mole^−1^. Subscript 1 refers to Zr(*α*). Subscript 2 refers to H_2_(g).

*T*	Phases present	$\frac{H^{\circ }-H_{298.15}^{\circ }}{T}$	$C_{p}^{\circ }$	*S*°	$\frac{G^{{}^{\circ}}-H_{298.15}^{{}^{\circ}}}{T}$	$\frac{{\bar{H}}_{1}-H_{1}^{{}^{\circ}}}{T}$	${\left({\bar{C}}_{p}\right)}_{1}$	${\bar{S}}_{1}$	$\frac{{\bar{G}}_{1}-G_{1}^{{}^{\circ}}}{T}$	$\frac{{\bar{H}}_{2}-H_{2}^{{}^{\circ}}}{T}$	${\left({\bar{C}}_{p}\right)}_{2}$	${\bar{S}}_{2}$	$\frac{{\bar{G}}_{2}-G_{2}^{{}^{\circ}}}{T}$

298.15	*α+δ*	0	6.81	9.22	−9.22	0.02	6.32	9.31	−0.00	−139.12	1.71	−0.29	−107.62
300	*α+δ*	0.04	6.82	9.26	−9.22	.02	6.32	9.34	−.00	−138.29	1.74	−.28	−106.76
350	*α+δ*	1.04	7.24	10.34	−9.31	.02	6.42	10.33	−.00	−119.20	2.86	+.07	−86.95
400	*α+δ*	1.85	7.79	11.35	−9.50	.02	6.58	11.19	−.00	−104.73	4.24	.54	−72.02
450	*α+δ*	2.54	8.40	12.30	−9.76	.03	6.81	11.98	−.00	−93.32	5.57	1.12	−60.36
500	*α+δ*	3.16	8.99	13.22	−10.06	.04	7.12	12.71	−.00	−84.07	6.56	1.76	−51.03
550	*α+δ*	3.71	9.52	14.10	−10.39	.08	7.51	13.41	−.01	−76.44	7.05	2.41	−43.38
600	*α+δ*	4.22	9.98	14.95	−10.73	.13	7.97	14.08	−.02	−70.06	7.03	3.03	−37.01
650	*α+δ*	4.68	10.40	15.76	−11.09	.21	8.48	14.74	−.04	−64.69	6.75	3.58	−31.62
700	*α+δ*	5.10	10.94	16.55	−11.45	.31	9.01	15.39	−.06	−60.09	6.76	4.08	−27.00
750	*α+δ*	5.52	11.81	17.33	−11.82	.43	9.53	16.03	−.08	−56.07	8.00	4.58	−23.00
800	*α+δ*	5.96	13.38	18.14	−12.18	.55	10.00	16.66	−.11	−52.41	11.86	5.20	−19.49
820	*α+δ*	6.15	14.31	18.48	−12.34	.61	10.17	16.91	−.13	−50.98	14.54	5.52	−18.22
820	*β*	8.85	10.89	21.19	−12.34	2.21	9.66	18.51	−.13	−47.10	4.29	9.41	−18.22
850	*β*	8.93	10.89	21.58	−12.65	2.21	9.66	18.86	−.21	−45.53	4.29	9.56	−16.55
900	*β*	9.04	10.89	22.20	−13.17	2.20	9.66	19.41	−.34	−43.16	4.29	9.80	−14.02
950	*β*	9.13	10.89	22.79	−13.66	2.19	9.66	19.93	−.45	−41.04	4.29	10.03	−11.74
1000	*β*	9.22	10.89	23.35	−14.13	2.18	9.66	20.42	−.56	−39.14	4.29	10.25	−9.69
1050	*β*	9.30	10.89	23.88	−14.58	2.15	9.66	20.90	−.67	−37.41	4.29	10.46	−7.82
1100	*β*	9.37	10.89	24.39	−15.02	2.12	9.66	21.35	−.77	−35.85	4.29	10.66	−6.12
1136	*β*	9.42	10.89	24.74	−15.32	2.09	9.66	21.66	−.84	−34.80	4.29	10.80	−4.98
1150	*β*	9.44	10.89	24.87	−15.43	2.08	9.66	21.78	−.86	−34.42	4.29	10.86	−4.56
1200	*β*	9.50	10.89	25.33	−15.84	2.03	9.66	22.19	−.95	−33.11	4.29	11.04	−3.12

**Table 12:** Thermodynamic functions for *ZrH_0.75_* *T* in deg K, thermodynamic functions in cal (deg K)^−1^ mole^−1^. Subscript 1 refers to Zr(*α*). Subscript 2 refers to H_2_(g).

*T*	Phases present	$\frac{H^{\circ }-H_{298.15}^{\circ }}{T}$	$C_{p}^{\circ }$	*S*°	$\frac{G^{{}^{\circ}}-H_{298.15}^{{}^{\circ}}}{T}$	$\frac{{\bar{H}}_{1}-H_{1}^{{}^{\circ}}}{T}$	${\left({\bar{C}}_{p}\right)}_{1}$	${\bar{S}}_{1}$	$\frac{{\bar{G}}_{1}-G_{1}^{{}^{\circ}}}{T}$	$\frac{{\bar{H}}_{2}-H_{2}^{{}^{\circ}}}{T}$	${\left({\bar{C}}_{p}\right)}_{2}$	${\bar{S}}_{2}$	$\frac{{\bar{G}}_{2}-G_{2}^{{}^{\circ}}}{T}$

298.15	*α+δ*	0	6.97	9.20	−9.20	0.02	6.32	9.31	−0.00	−139.12	1.71	−0.29	−107.62
300	*α+δ*	0.04	6.98	9.24	−9.20	.02	6.32	9.34	−.00	−138.29	1.74	−.28	−106.76
350	*α+δ*	1.07	7.49	10.35	−9.28	.02	6.42	10.33	−.00	−119.20	2.86	+ .07	−86.95
400	*α+δ*	1.91	8.17	11.40	−9.48	.02	6.58	11.19	−.00	−104.73	4.24	.54	−72.02
450	*α+δ*	2.65	8.90	12.40	−9.75	.03	6.81	11.98	−.00	−93.32	5.57	1.12	−60.36
500	*α+δ*	3.31	9.58	13.37	−10.06	.04	7.12	12.71	−.00	−84.07	6.56	1.76	−51.03
550	*α+δ*	3.91	10.16	14.32	−10.41	.08	7.51	13.41	−.01	−76.44	7.05	2.41	−43.38
600	*α+δ*	4.45	10.61	15.22	−10.77	.13	7.97	14.08	−.02	−70.06	7.03	3.03	−37.01
650	*α+δ*	4.94	11.01	16.08	−11.15	.21	8.48	14.74	−.04	−64.69	6.75	3.58	−31.62
700	*α+δ*	5.39	11.55	16.92	−11.53	.31	9.01	15.39	−.06	−60.09	6.76	4.08	−27.00
750	*α+δ*	5.83	12.53	17.74	−11.92	.43	9.53	16.03	−.08	−56.07	8.00	4.58	−23.00
800	*α+δ*	6.30	14.45	18.61	−12.31	.55	10.00	16.66	−.11	−52.41	11.86	5.20	−19.49
820	*α+δ*	6.51	15.62	18.98	−12.47	.61	10.17	16.91	−.13	−50.98	+14.54	+5.52	−18.22
820	*α+β*	8.41	23.55	20.87	−12.47	5.88	33.12	22.19	−.13	−60.00	−25.52	−3.50	−18.22
850	*α+β*	8.98	25.08	21.76	−12.78	6.56	31.21	23.35	−.35	−58.86	−16.36	−4.24	−16.08
891.5	*α+β*	9.71	23.77	22.93	−13.22	7.25	26.40	24.73	−.68	−56.99	−7.03	−4.79	−13.32
891.5	*β*	9.71	11.27	22.93	−13.22	2.20	9.66	19.68	−.68	−43.55	+4.29	+8.65	−13.32
900	*β*	9.72	11.27	23.04	−13.32	2.20	9.66	19.78	−.70	−43.16	4.29	8.69	−12.91
950	*β*	9.80	11.27	23.65	−13.84	2.19	9 66	20.30	−.82	−41.04	4.29	8.92	−10.63
1000	*β*	9.88	11 27	24.22	−14.35	2.18	9.66	20.80	−.94	−39.14	4.29	9.14	−8.58
1050	*β*	9.94	11.27	24.78	−14.83	2.15	9.66	21.27	−1.04	−37.41	4.29	9.35	−6.71
1100	*β*	10.00	11.27	25.30	−15.30	2.12	9.66	21.72	−1.14	−35.85	4.29	9.55	−5.00
1136	*β*	10.04	11.27	25.66	−15.62	2.09	9.66	22.03	−1.21	−34.80	4.29	9.69	−3.87
1150	*β*	10.06	11.27	25.80	−15.74	2.08	9.66	22.15	−1.23	−34.42	4.29	9.75	−3.44
1200	*β*	10.11	11.27	26.28	−16.17	2.03	9.66	22.56	−1.32	−33.11	4.29	9.93	−2.01

**Table 13:** Thermodynamic functions for ZrH_1.00_ *T* in deg K, thermodynamic functions in cal (deg K)^−1^ mole^−1^. Subscript 1 refers to Zr(*α*). Subscript 2 refers to H_2_(g).

*T*	Phases present	$\frac{H^{\circ }-H_{298.15}^{\circ }}{T}$	$C_{p}^{\circ }$	*S*°	$\frac{G^{{}^{\circ}}-H_{298.15}^{{}^{\circ}}}{T}$	$\frac{{\bar{H}}_{1}-H_{1}^{{}^{\circ}}}{T}$	${\left({\bar{C}}_{p}\right)}_{1}$	${\bar{S}}_{1}$	$\frac{{\bar{G}}_{1}-G_{1}^{{}^{\circ}}}{T}$	$\frac{{\bar{H}}_{2}-H_{2}^{{}^{\circ}}}{T}$	${\left({\bar{C}}_{p}\right)}_{2}$	${\bar{S}}_{2}$	$\frac{{\bar{G}}_{2}-G_{2}^{{}^{\circ}}}{T}$

298.15	*α+δ*	0	7.18	9.16	−9.16	0.02	6.32	9.31	−0.00	−139.12	1.71	−0.29	−107.62
300	*α+δ*	0.04	7.20	9.20	−9.16	.02	6.32	9.34	−.00	−138.29	1.74	−.28	−106.76
350	*α+δ*	1.11	7.85	10.36	−9.25	.02	6.42	10.33	−.00	−119.20	2.86	+ .07	−86.95
400	*α+δ*	2.00	8.70	11.46	−9.46	.02	6.58	11.19	−.00	−104.73	4.24	.54	−72.02
450	*α+δ*	2.80	9.60	12.54	−9.74	.03	6.81	11.98	−.00	−93.32	5.57	1.12	−60.36
500	*α+δ*	3.52	10.40	13.59	−10.07	.04	7.12	12.71	−.00	−84.07	6.56	1.76	−51.03
550	*α+δ*	4.18	11.04	14.62	−10.44	.08	7.51	13.41	−.01	−76.44	7.05	2.41	−43.38
600	*α+δ*	4.77	11.49	15.60	−10.83	.13	7.97	14.08	−.02	−70.06	7.03	3.03	−37.01
650	*α+δ*	5.30	11.85	16.53	−11.23	.21	8.48	14.74	−.04	−64.69	6.75	3.58	−31.62
700	*α+δ*	5.78	12.39	17.43	−11.64	.31	9.01	15.39	−.06	−60.09	6.76	4.08	−27.00
750	*α+δ*	6.26	13.53	18.32	−12.06	.43	9.53	16.03	−.08	−56.07	8.00	4.58	−23.00
800	*α+δ*	6.78	15.93	19.26	−12.48	.55	10.00	16.66	−.11	−52.41	11.86	5.20	−19.49
820	*α+δ*	7.02	17.44	19.67	−12.65	.61	10.17	16.91	−.13	−50.98	+ 14.54	+5.52	−18.22
820	*β+δ*	7.79	20.36	20.44	−12.65	5.88	33.12	22.19	−.13	−60.00	−25.52	−3.50	−18.22
850	*β+δ*	8.29	23.04	21.23	−12.94	6.56	31.21	23.35	−.35	−58.86	−16.36	−4.24	−16.08
900	*β+δ*	9.11	22.60	22.55	−13.44	7.35	25.37	24.97	−.75	−56.58	−5.52	−4.85	−12.78
950	*β+δ*	9.77	20.60	23.72	−13.95	7.75	20.07	26.19	−1.16	−54.08	+1.07	−4.96	−9.79
1000	*β+δ*	10.27	19.04	24.73	−14.46	7.89	16.68	27.13	−1.56	−51.58	4.72	−4.80	−7.08
1050	*β+δ*	10.66	17.99	25.63	−14.97	7.88	14.76	27.89	−1.94	−49.20	6.47	−4.52	−4.62
1080.9	*β+δ*	10.86	17.46	26.15	−15.28	7.83	13.97	28.31	−2.17	−47.80	6.98	−4.32	−3.22
1080.9	*β*	10.86	11.81	26.15	−15.28	2.13	9.66	22.62	−2.17	−36.43	4.29	+7.06	−3.22
1100	*β*	10.88	11.81	26.36	−15.48	2.12	9.66	22.79	−2.21	−35.85	4.29	7.14	−2.59
1136	*β*	10.91	11.81	26.74	−15.83	2.09	9.66	23.10	−2.28	−34.80	4.29	7.28	−1.45
1150	*β*	10.92	11.81	26.88	−15.96	2.08	9.66	23.22	−2.30	−34.42	4.29	7.33	−1.03
1200	*β*	10.96	11.81	27.38	−16.42	2.03	9.66	23.63	−2.39	−33.11	4.29	7.52	+0.41

**Table 14:** Thermodynamic functions for *ZrH_1.25_* *T* in deg K, thermodynamic functions in cal (deg K)^−1^ mole^−1^. Subscript 1 refers to Zr(*α*). Subscript 2 refers to H_2_(g).

*T*	Phases present	$\frac{H^{\circ }-H_{298.15}^{\circ }}{T}$	$C_{p}^{\circ }$	*S*°	$\frac{G^{{}^{\circ}}-H_{298.15}^{{}^{\circ}}}{T}$	$\frac{{\bar{H}}_{1}-H_{1}^{{}^{\circ}}}{T}$	${\left({\bar{C}}_{p}\right)}_{1}$	${\bar{S}}_{1}$	$\frac{{\bar{G}}_{1}-G_{1}^{{}^{\circ}}}{T}$	$\frac{{\bar{H}}_{2}-H_{2}^{{}^{\circ}}}{T}$	${\left({\bar{C}}_{p}\right)}_{2}$	${\bar{S}}_{2}$	$\frac{{\bar{G}}_{2}-G_{2}^{{}^{\circ}}}{T}$

298.15	*α+δ*	0	7.39	9.12	−9.12	0.02	6.32	9.31	−0.00	−139.12	1.71	−0.29	−107.62
300	*α+δ*	0.05	7.42	9.17	−9.12	.02	6.32	9.34	−.00	−138.29	1.74	−.28	−106.76
350	*α+δ*	1.15	8.21	10.37	−9.22	.02	6.42	10.33	−.00	−119.20	2.86	+.07	−86.95
400	*α+δ*	2.10	9.23	11.53	−9.34	.02	6.58	11.19	−.00	−104.73	4.24	.54	−72.02
450	*α+δ*	2.95	10.29	12.68	−9.73	.03	6.81	11.98	−.00	−93.32	5.57	1.12	−60.36
500	*α+δ*	3.73	11.23	13.81	−10.08	.04	7.12	12.71	−.00	−84.07	6.56	1.76	−51.03
550	*α+δ*	4.45	11.92	14.92	−10.47	.08	7.51	13.41	−.01	−76.44	7.05	2.41	−43.38
600	*α+δ*	5.09	12.37	15.98	−10.89	.13	7.97	14.08	−.02	−70.06	7.03	3.30	−37.01
650	*α+δ*	5.66	12.70	16.98	−11.32	.21	8.48	14.74	−.04	−64.69	6.75	3.58	−31.62
700	*α+δ*	6.18	13.24	17.94	−11.76	.31	9.01	15.39	−.06	−60.09	6.76	4.08	−27.00
750	*α+δ*	6.69	14.53	18.89	−12.20	.43	9.53	16.03	−.08	−56.07	8.00	4.58	−23.00
791.5	*α+δ*	7.15	16.77	19.73	−12.57	.53	9.93	16.55	−.11	−53.02	10.94	5.08	−20.05
791.5	*δ*	7.15	13.12	19.73	−12.57	2.91	13.12	18.94	−.11	−56.82	0.00	1.26	−20.05
800	*δ*	7.22	13.12	19.87	−12.65	2.94	13.12	19.08	−.15	−56.29	.00	1.26	−19.44
820	*δ*	7.36	13.12	20.19	−12.83	3.01	13.12	19.40	−.22	−55.09	.00	1.26	−18.07
845.6	*δ*	7.53	13.12	20.59	−13.06	3.09	13.12	19.80	−.32	−53.64	.00	+1.26	−16.39
845.6	*β+δ*	7.53	20.64	20.59	−13.06	6.49	31.62	23.18	−.32	−59.05	−17.56	−4.16	−16.39
850	*β+δ*	7.60	20.99	20.70	−13.10	6.56	31.21	23.35	−.35	−58.86	−16.36	−4.24	−16.08
900	*β+δ*	8.39	21.91	21.94	−13.55	7.35	25.37	24.97	−.75	−56.58	−5.52	−4.85	−12.78
950	*β+δ*	9.07	20.74	23.10	−14.02	7.75	20.07	26.19	−1.16	−54.08	+1.07	−4.96	−9.79
1000	*β+δ*	9.63	19.63	24.13	−14.50	7.89	16.68	27.13	−1.56	−51.58	4.72	−4.80	−7.08
1050	*β+δ*	10.08	18.80	25.07	−14.99	7.88	14.76	27.89	−1.94	−49.20	6.47	−4.52	−4.62
1100	*β+δ*	10.46	18.03	25.93	−15.46	7.78	13.56	28.55	−2.31	−46.98	7.15	−4.20	−2.38
1136	*β+δ*	10.69	17.45	26.50	−15.81	7.68	12.87	28.98	−2.56	−45.49	7.32	−3.97	−0.89
1150	*β+δ*	10.77	17.22	26.71	−15.94	7.64	12.62	29.13	−2.66	−44.94	7.35	−3.88	−.34
1200	*β+δ*	11.02	16.39	27.42	−16.40	7.47	11.76	29.65	−2.98	−43.06	7.41	−3.56	+1.53

**Table 15:** Ideal-gas thermodynamic junctions for normal hydrogen, *H_2_* (25 percent para, 75 percent ortho) *T* in deg K, thermodynamic functions in cal (deg K)^−1^ mole^−1^

*T*	$\frac{H^{\circ }-H_{298.15}^{\circ }}{T}$	$C_{p}^{\circ }$	*S*°	$\frac{G^{{}^{\circ}}-H_{298.15}^{{}^{\circ}}}{T}$

298.15	0	6.891	31.209	−31.209
300	0.043	6.894	31.252	−31.209
350	1.026	6.951	32.321	−31.295
400	1.769	6.975	33.251	−31.482
450	2.347	6.987	34.072	−31.725
500	2.813	6.993	34.809	−31.996
550	3.192	7.001	35.477	−32.285
600	3.511	7.009	36.085	−32.574
650	3.780	7.021	36.617	−32.867
700	4.011	7.037	37.168	−33.157
750	4.214	7.057	37.653	−33.439
791.5	4.363	7.077	38.034	−33.671
800	4.392	7.081	38.110	−33.718
820	4.458	7.092	38.285	−33.827
845.6	4.537	7.108	38.502	−33.965
850	4 551	7.110	38.539	−33.988
865.3	4.596	7.120	38.666	−34.070
891.5	4.671	7.136	38.879	−34.208
900	4.695	7.142	38.946	−34.251
950	4.825	7.180	39.334	−34.509
956.6	4.841	7.185	39 .384	−34.543
1000	4.944	7.220	39 .702	−34.758
1050	5.052	7.201	40.055	−35.003
1080.9	5.116	7.291	40 .267	−35.151
1100	5.154	7.309	40.395	−35.241
1136	5.223	7.343	40 .631	−35.408
1150	5.249	7.357	40 .721	−35.472
1200	5.337	7.406	41.035	−35.698

## Footnotes

1 Figures in brackets indicate the literature references at the end of this paper.

2 Some authors have used “*γ*” in place of “*δ*” in this system, *“γ,”* however, has come to be used [3,12] to designate a type of metastable phase in the system not discussed in the present paper.

3 Face-centered cubic may here be considered a special case of face-centered tetragonal. By a simple rotation of crystal axes, the *δ* face-centered tetragonal lattice may be transformed into body-centered tetragonal (with a different unit cell), for comparison with the *β* body-centered cubic lattice.

4 This convention seemed more logical than one based on mole fractions, since it is probable that hydrogen (as atoms) enters the zirconium lattices interstitially (with vacancies) instead of substitutionally.

5 By this convention one mole of ZrH*_x_* thus contains one mole of Zr and ½ *x* moles of the component H_2_. The conversion to standard states involving *β*-Zr and atomic H could of course readily be made if desired.

6 It as assumed in eq (26) and related eqs (30–36, 39, 40) that the values of *x* lie in the (*α*+*β*) field at 298 °K.

7 In an earlier paper [17] the author defined an equivalent quantity and called ti the “adjusted heat content.”

8 Even if the eutectoid temperature is really as low as 820 °K (eq (46)), the (*α*+*δ*) enthalpy isotherm at 823 °K is not to be interpreted as invalid, since the samples were not heated above 823 °K in these measurements and the *β* phase could easily have failed to appear.

9 Equation (51) agrees exactly with table 3. However, owing to a small computation error, eq (50) differs slightly from the table above 473 °K (by an average of 7 cal mole^−1^, which is within the precision of the data).

10 The values in the second column of the table are sensitive to the power of *x* assumed. With the assumption of the 820 °K value as before, the second column was recalculated with $a_{2}^{\alpha }$ proportional to *x*^1.75^, but the disagreement with the third column was much worse. It may be noted, however, that the extent of disagreement is independent ol the value assumed for the eutectoid hydrogen activity, for the same value was assumed in deriving eqs (69) and (70), and cancels when the two equations are set equal to each other.

11 Equation (82) follows also from eqs (11) and (81). Both equations (81) and (82) are consistent with the assumption made earlier that the *α* phases are ideally dilute solutions of atomic hydrogen in *α*-zirconium.

12 The composition *x*=0.556 in table 5, while outside the range to which eq (92) has been restricted, is too close to *x*=0.57 to invalidate appreciably the differences “observed minus calculated” noted in the table.

13 The tolerances attached to two of the values in the table represent not precision or estimated accuracy, but the uncertainty due to hysteresis. For these two points the temperature was approached both by heating and by cooling (in two separate series of runs). The enthalpies found were higher in the former than in the latter series by twice the stated tolerance, and the mean of the two series has been tabulated.

14 The discrepancies are much larger than those found in several tests of heating- and-cooling hysteresis, and seem to be due to different locations of the *β*/(*β*+*δ*) boundary caused by different impurities in the two samples.

15 The values indicated by the pressure measurements of Gulbransen and Andrew |4] and of Ells and McQuillan [6] seem to fall within a range of ±10 percent of this value.

16 For the eutectoid *δ* phase *(x*= 1.171, *T*=820), eq (134) should of course give the same value of *S*° as eqs (5), (79), and (80) for the *(α+δ*) field. The two values calculated were found to differ by only 0.002. However, this is a check of thermodynamic consistency only.

17 Relatively accentuated for higher values of r in figure 1 compared with the more usual plots of the phase diagram as temperature versus *atomic percentage* of hydrogen.

18 This curvature of the *δ*-field isotherms increases the difficulty of locating the compositions of the breaks accurately, and suggests that some of the values of *x* selected in the references cited may be somewhat too high.

19 See footnote 13 (table 5).

20 Douglas and Victor’s value of 9.0 for *β*-Zr, which is greater than they found for ZrH_0.324_, is not precise because of the short temperature range, and may be too high if the small amount of oxygen and nitrogen (0.065 atom %) raised the transformation temperature above 1,153 °K [6].

21 Ells and McQuillan [6] concluded, e.g., that oxygen as an impurity raises this boundary.

22 The boundaries are insensitive to the eutectoid hydrogen pressure used as an integration constant.

23 By retaining the values of eq (138), the enthalpies of ZrH_0.324_ below 1,173 °K were varied by many times what seems the probable uncertainty of the observed values. Mow ever, this procedure had the desired effect of increasing (∂*H^β^/∂x*)*_T_* at the lower temperatures near the phase-field boundary.

24 As a further check, the values of $\left({\bar{H}}_{1}^{\alpha +\beta }-H_{1}^{\circ }\right)/T$ and $\left({\bar{H}}_{2}^{\alpha +\beta }-H_{2}^{\circ }\right)/T$ agreed approximately with those estimated from eq (11).

## References

[1] Sieverts A, Roell E (1926). Zirconium, thorium, and hydrogen. Z anorg allgem Chem.

[2] Hall MNA, Martin SLH, Rees ALG (1945). Solubility of hydrogen in zirconium and zirconium- oxygen solid solutions. Trans Faraday Soc.

[3] Gulbransen EA, Andrew KF (1954). Crystal structure and thermodynamic studies on the zirconium-hydrogen alloys. J Electrochem Soc.

[4] Gulbransen EA, Andrew KF (1955). Solubility and decomposition pressures of hydrogen in alpha-zirconium. J Metals.

[5] Edwards RK, Levesque P, Cubicciotti D (1955). Solid solution equilibria in the zirconium-hydrogen system. J Amer Chem Soc.

[6] Ells CE, McQuillan AD (1956). A study of the hydrogen-pressure relationships in the zirconium-hydrogen system. J Inst Metals.

[7] Mallett MW, Albrecht WM (1957). Low-pressure solubility and diffusion of hydrogen in zirconium. J Electrochem Soc.

[8] LaGrange LD, Dykstra LJ, Dixon JM, Merten U (1959). A study of the zirconium-hydrogen and zirconium-hydrogen-uranium systems between 600 and 800°. J Phys Chem.

[9] Schwartz CM, Mallett MW (1954). Observations on the behavior of hydrogen in zirconium. Trans Amer Soc Metals.

[10] Vaughan DA, Bridge JR (1956). High-temperature X-ray diffraction investigation of the Zr-H system. J Metals.

[11] Rundie RE, Shull CG, Wollan EO (1952). The crystal structure of thorium and zirconium dihydrides by X-ray and neutron diffraction. Acta Cryst.

[12] Beck RL (1962). Thermophysical properties of zirconium hydride. Trans Amer Soc Metals.

[13] Espagno L, Azou P, Bastien P (1958). Dilatometrie study of the system zirconium-hydrogen. Compt rend.

[14] Espagno L, Azou P, Bastien P (1959). Dilatometrie study of the zirconium-hydrogen system between ambient and 500°. Compt rend.

[15] Flotow HE, Osborne DW (1961). Heat capacities and thermodynamic functions of ZrH_2_ and ZrD_2_ from 5 to 350°K and the hydrogen vibration frequency in ZrH_2_. J Chem Phys.

[16] Douglas TB, Victor AC (1958). Heat content of zirconium and of five compositions of zirconium hydride from 0 to 900 °C. J Research NBS.

[17] Douglas TB (1958). The zirconium-hydrogen system: some thermodynamic properties from a heat content study. J Amer Chem Soc.

[18] Martin SLH, Rees ALG (1954). Interpretation of the solubility of hydrogen in zirconium. Trans Faraday Soc.

[19] Libowitz GG (1960). The nature and properties of transition metal hydrides. J nuclear materials.

[20] Hilsenrath J, Beckett CW, Benedict WS, Fano L, Hoge HJ, Masi JF, Nuttall RL, Touloukian YS, Woolley HW (1955). Tables of thermal properties of gases, NBS Circ. 564.

[21] Rossini FD, Wagman DD, Evans WH, Levine S, Jaffe I (1952). Selected values of chemical thermodynamic properties, NBS Circ 500.

[22] Skinner GB (1951). Thermodynamic and structural properties of zirconium. PhD Dissertation.

[23] Scott JL (1957). A calorimetric investigation of zirconium, titanium, and zirconium alloys from 60 to 960° C, A.E.C. Report ORNL–2328.

[24] Todd SS (1950). Heat capacities at low temperatures and entropies of zirconium, zirconium nitride, and zirconium tetrachloride. J Am Chem Soc.

[25] Skinner GB, Johnston HL (1951). Low temperature heat capacities of inorganic solids. VIII. Heat capacity of zirconium from 14 to 300° K. J Am Chem Soc.

[26] Estermann I, Friedberg SA, Goldman JE (1952). The specific heats of several metals between 1.8° and 4.2° K. Phys Rev.

[27] Wolcott NM (1957). The atomic heats of titanium, zirconium, and hafnium. Phil Mag [8].

[28] Burk DL, Estermann I, Friedberg SA (1958). The low temperature specific heats of titanium, zirconium, and hafnium. Z physik Chem (Neue Folge, Frankfurt).

[29] Mixter WG, Dana ES (1873). Specific heats of zirconium, silicon, and boron. Liebigs Ann Chem.

[30] Jaeger FM, Veenstra WA (1934). The exact measurement of the specific heats of solid substances at high temperatures. VII. The calorimetric behavior of zirconium. Ree trav chim.

[31] Coughlin JP, King EG (1950). High-temperature heat contents of some zirconium-containing substances. J Am Chem Soc.

[32] Redmond RF, Lones J (1952). Enthalpies and heat capacities of stainless steel (316), zirconium, and lithium at elevated temperatures, A.E.C. Report ORNL-1342.

[33] Furukawa GT, Reilly ML (1961). National Bureau of Standards.

